# Sustainable and eco-friendly syntheses of green MXenes for advanced battery applications

**DOI:** 10.1186/s40580-025-00504-2

**Published:** 2025-07-26

**Authors:** Seonju Kim, Hyeonmin Jo, Jiyoung Yun, Jun-Won Lee, Jiung Cho, Kisuk Kang, Hee-Dae Lim

**Affiliations:** 1 Department of Chemical Engineering, Hanyang University, Seoul, 04763 Republic of Korea; 2 Department of Materials Science and Engineering, Hongik University, Sejong, Republic of Korea; 3 Department of Materials Science and Engineering, Seoul National University, Seoul, 08826 Republic of Korea; 4 Department of Battery Engineering, Hanyang University, Seoul, 04763 Republic of Korea

**Keywords:** MXene, Batteries, Fluorine-free, Green synthesis, Sustainable materials

## Abstract

MXenes, a versatile family of two-dimensional (2D) transition metal carbides and nitrides, have attracted significant attention for battery applications due to their exceptional properties, such as high electronic conductivity, tunable microstructure, robust mechanical and chemical stability, and compositional diversity. However, despite these advantages, conventional MXene synthesis methods-relying heavily on toxic acid etching**-**pose serious environmental hazards, undermining their suitability for sustainable energy applications. In this context, eco-friendly and non-toxic MXene synthesis routes have become increasingly critical for enabling the widespread adoption of MXene, driving extensive research into alternative, green synthetic approaches. These recent advances in environmentally benign synthesis are pivotal to unlocking the full potential of MXenes for diverse next-generation battery technologies. In this review, we provide a comprehensive overview of green and sustainable MXene synthesis strategies, highlighting the latest developments that go beyond traditional fluorine-based routes. Each synthetic process is comparatively analyzed with respect to its efficacy, limitations, and implications for practical application as key functional components in lithium-ion batteries (LIBs) and post-LIB systems. Finally, we offer a perspective on how the development of eco-friendly MXenes can contribute to overcoming the industrial challenges facing advanced battery technologies.

**Graphical Abstract:**

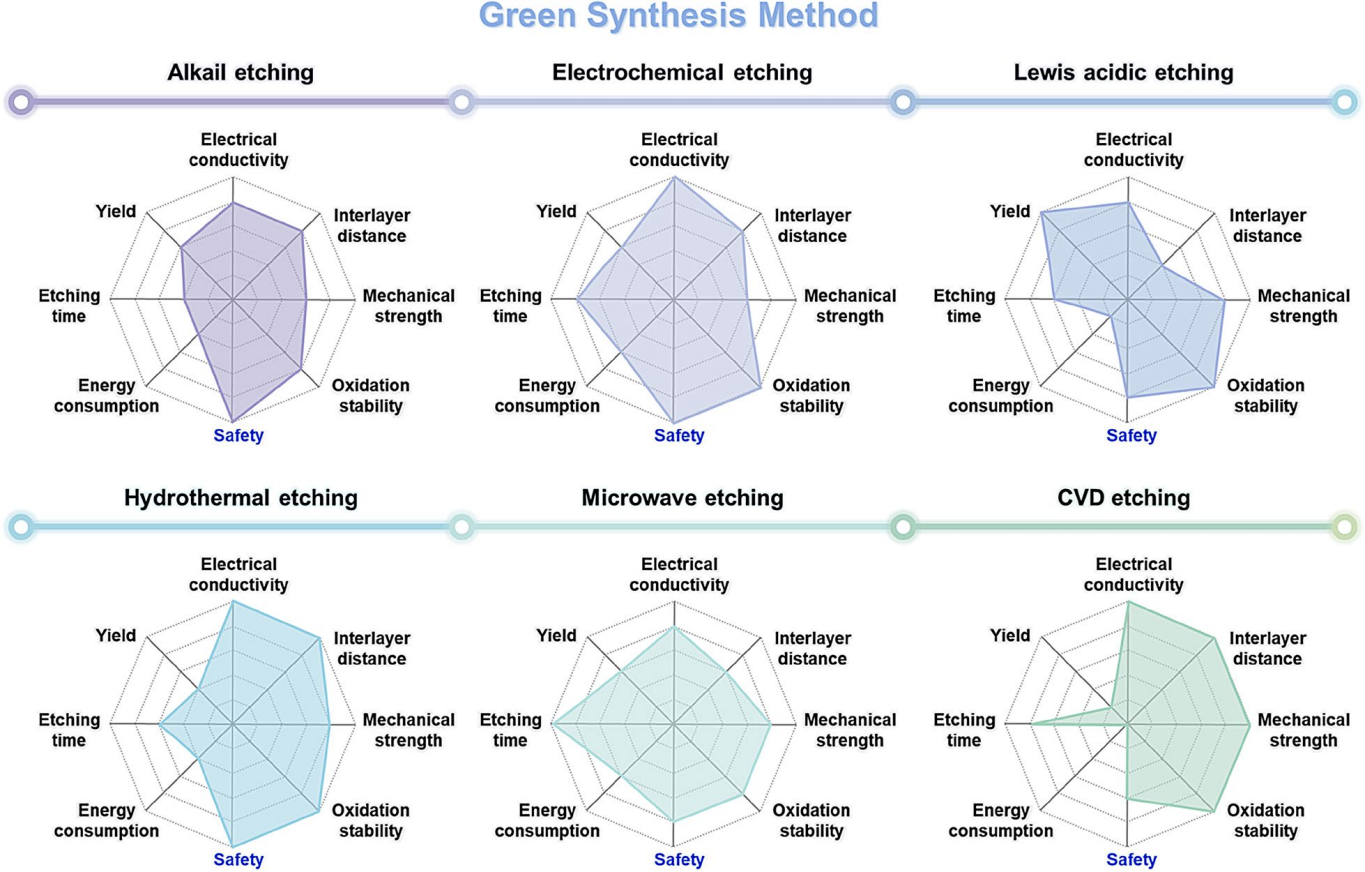

## Introduction

With the global transition toward electric mobility and renewable energy integration, the demand for high-performance, cost-effective and safer rechargeable batteries continue to rise. Li-ion batteries (LIBs) are at the frontline in this transformation and have undergone remarkable technological advancements, for example, with energy densities increasing from 90 to ~ 300 Wh kg^− 1^ over the past decades [1–3]. Despite their continued dominance, LIBs are facing growing challenges in meeting the evolving requirements for higher energy density, longer cycle life, and reduced manufacturing costs [4, 5]. Addressing these issues has spurred extensive research into next-generation electrode materials, the key performance and cost factors of LIBs, including high-capacity cathodes [6–9], silicon-based anodes [10–12], and Li metal anodes [13–16]. For instance, Li metal anodes can boost energy density close to ~ 500 Wh kg^− 1^ [17], pairing them with sulfur cathodes may push this further to ~ 600 Wh kg^− 1^ at the cell level [18]. Silicon-based anodes also offer theoretical capacities nearly ten times greater than conventional graphite anodes, presenting a compelling route to achieve higher energy densities [19].

Nevertheless, the integration of such advanced materials into practical battery systems remains hindered by issues such as large volume changes, unstable electrode-electrolyte interfaces and rapid material degradation [20–22]. One of the feasible ways to tackle these challenges has been to employ functional additives, including conducting carbon buffers [23–25], artificial interlayers [26, 27], and electrolyte additives [28, 29]. These additives could facilitate uniform ion transport, enhance interfacial stability, and reduce cell impedance, thereby extending cycling stability [30, 31]. Additionally, improving the performance and reducing the content of inactive battery components, such as binders, conductive agents, and separators, has emerged as an effective route to maximizing cell-level energy density. While the practical development of active electrode materials remains stalled, minimizing the content of these components, optimizing their functionality, and introducing new functional materials present effective strategy. As a representative example, multi-wall or single-wall carbon nanotubes (CNTs) have been adopted in commercial batteries due to their superior electrical conductivity and mechanical strength compared to traditional carbon blacks [32–34]. Electrolyte additives such as fluoroethylene carbonate (FEC) and LiNO_3_ are also known to promote the formation of more resilient SEI (solid-electrolyte interphase) layers on lithium metal anodes, contributing to improved stability and Coulombic efficiency [35, 36]. These functional additives offer substantial practical benefits, enhancing performance with minimal disruption to the overall battery chemistry and without imposing significant processing or cost burdens.

**Fig. 1:**
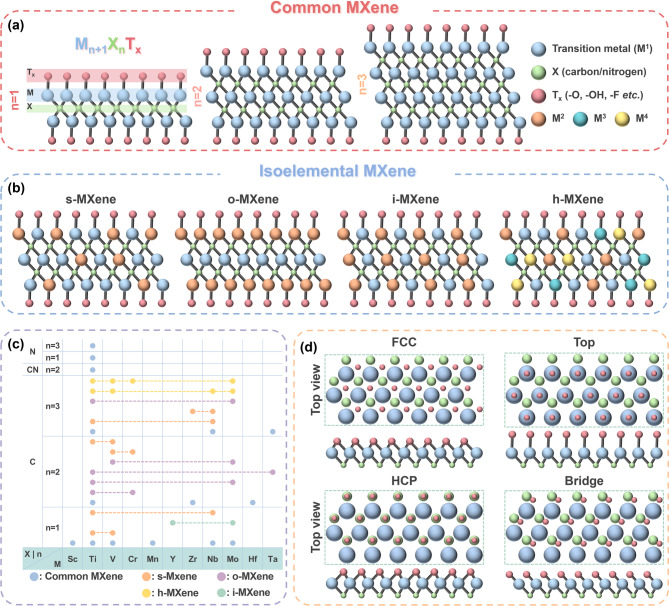
The structural and chemical diversity of MXenes. (**a**) Schematic illustration of layered structure of common MXenes with *n* = 1–3. (**b**) Schematic representation of general structure and classification of isoelemetal MXenes. (**c**) Diversity of MXenes based on various transition metals. (**d**) Top and side views of the four potential sites of surface terminations

Among emerging functional additives, MXenes–a family of two-dimensional transition metal carbides and nitrides–have recently gained considerable attention due to their tunable physicochemical properties. MXenes exhibit a unique combination of high electrical conductivity, hydrophilicity, chemical versatility, and mechanical integrity, making them highly attractive for a wide range of battery-related applications [37, 38]. The exploration of MXenes from diverse methods and chemistries has growingly unlocked the potential of MXenes as a key functional component in LIBs and post-LIB systems. Their general chemical formula, M_n+1_X_n_T_x_ (*n* = 1–3), includes an early transition metal (M), carbon and/or nitrogen layers (X), and surface terminations (T_x_) such as–O,–OH, and–F (Fig. 1a). MXenes are typically synthesized *via* selective etching of A-group elements (e.g., Al, Ga, In) from the parent MAX (M = transition metal, A = group 13 or 14 element, X = C or N) phase. Historically, this process has relied on harsh fluorine-containing etchants, particularly hydrofluoric acid (HF) to remove the A-layer and produce functional MXene sheets [39].

Despite the promising potential of MXenes in battery systems, the conventional synthesis routes pose severe limitations for industrial use. HF-based methods introduce significant environmental and safety hazards, presenting handling risks, and raises concerns about scalability and cost. These issues have prompted a growing need for alternative synthesis strategies that are safer, more sustainable, and commercially viable. In this context, the development of green, HF-free MXene synthesis methods has emerged as a critical research frontier. Such environmentally benign approaches aim to retain the functional advantages of MXenes, while eliminating the toxic byproducts and complex waste management requirements associated with traditional methods.

In this review, we present a comprehensive overview of green MXene synthesis techniques, tracing their historical development and evaluating key advances beyond fluorine-based approaches. We provide comparative analysis of various HF-free methods in terms of efficiency, achievable properties, and scalability. Furthermore, we highlight recent progress in applying green-synthesized MXenes to both LIBs and post-LIB systems, emphasizing their roles as multifunctional additives. Finally, we offer a perspective on how sustainable MXene technologies could address key industrial challenges and accelerate the adoption of next-generation battery solutions.

## General structure of MXene

MXenes can be classified into a few categories based on the arrangement and diversity of the M layers (Fig. 1b). The simplest category includes common MXenes with a basic chemical formula, containing a single type of M element with T_x_ terminations. The most popular form is Ti_3_C_2_T_x_ [39], while similar analogs such as Ti_2_CT_x_ [40] and V_2_CT_x_ [41] have also been studied. Isostructural MXenes incorporating at least two types of M elements have also been developed [42], which can be grouped in four subtypes based on their structural configuration (Fig. 1b): (1) random solid-solution MXene (*s*–MXene) [43], which contains a mixture of different M elements without a specific arrangement; (2) out-of-plane ordered MXenes (*o*–MXene) [44], wherein M elements are arranged in distinct layers along the *c*-axis; (3) in-plane ordered MXenes (*i*–MXene) [45], wherein alternating M elements are positioned laterally within the same atomic plane, and (4) high-entropy MXenes (*h*–MXene) [46], which are composed of multiple transition metals. Figure 1c illustrates how the chemical diversity of MXenes has been demonstrated with different components, wherein *x*- and *y*-axes represent various M and X elements. The dotted lines indicate the combinations of elements used when multiple M elements are incorporated. Apart from the diversity of M elements, several terminations exist in addition to the basic ones (–O,–OH, and–F), such as halogen terminations (–Cl,–Br, and–I), chalcogen terminations (–S,–Se, and–Te), and others (–Sb,–NH, etc.). Four potential termination sites on the MXene surface have been commonly considered, referred to as FCC, top, HCP, and bridge sites depending on their relative position with M and X layers, as illustrated in Fig. 1d. Computational studies have indicated that the surface termination located at the FCC site is energetically favored in many cases, while additional alternative sites may exist when the termination group is large [47]. This structural flexibility, combined with its chemical versatility, enables fine-tuning of MXene properties for a wide range of applications, with over 100 MXene compositions identified to date.

## Evolution of MXene synthesis methods: A historical perspective

Since its initial development, MXene synthesis has evolved rapidly, shifting from hazardous HF-based routes to more sustainable and scalable methods. To provide a clear synthesis-oriented perspective, we categorize these approaches into two main groups: (3.1) conventional synthesis methods, which typically involve toxic, HF-based etching techniques, and (3.2) green synthesis methods, which emphasize newly developed, environmentally friendly, and HF-free alternatives. In this section, we systematically examine key advancements within each category, highlighting their defining characteristics and tracing their historical evolution.

### Conventional synthesis methods

#### HF-based etching method

**Fig. 2:**
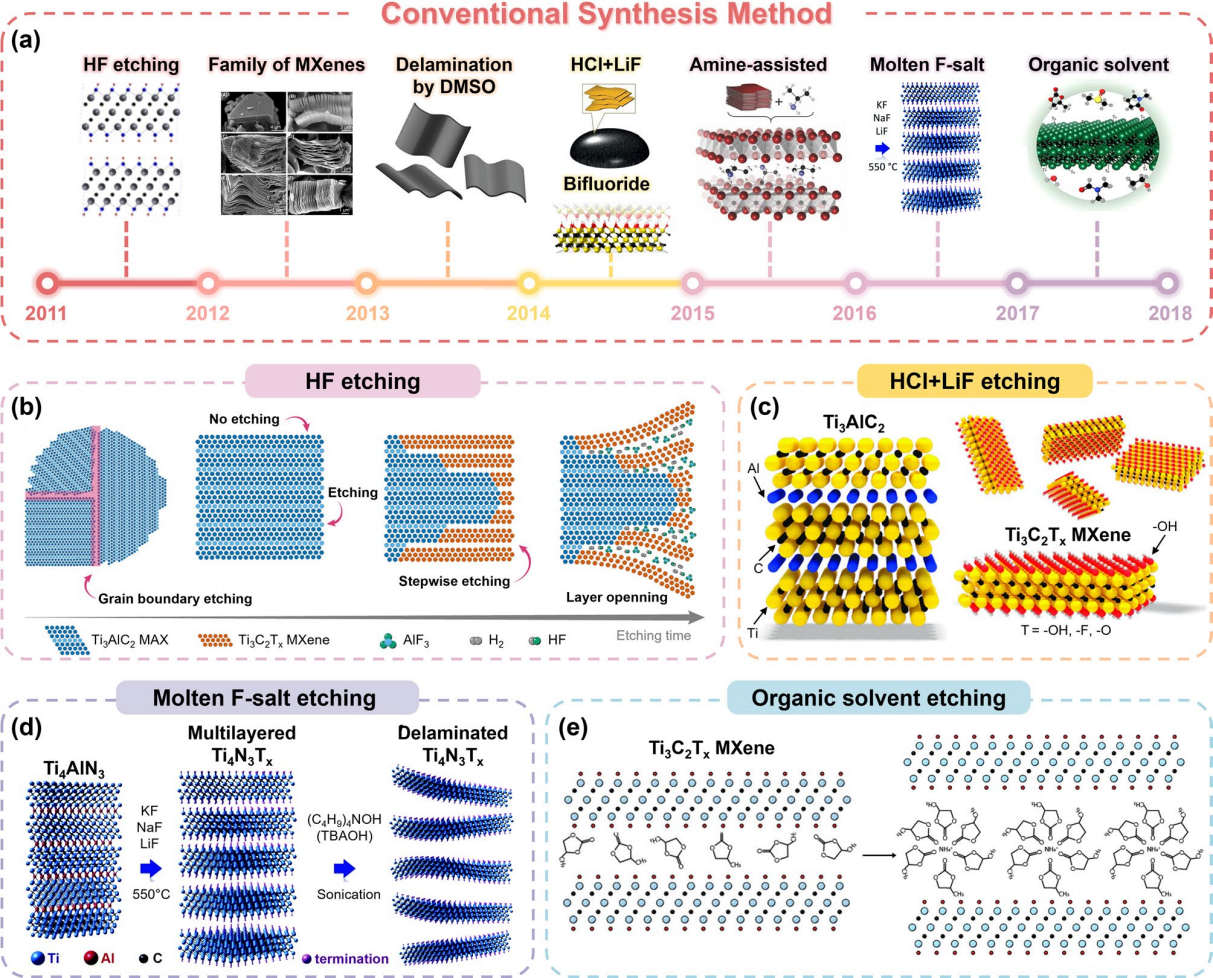
(**a**) Timeline illustrating the progress of Conventional Synthesis Methods, highlighting key advancements over time. Reproduced with permission from Ref [39, 65]. Copyright 2011, 2016, John Wiley & Sons, Inc. Reproduced with permission from Ref [49, 74, 75]. Copyright 2012, 2017, 2014, American Chemical Society. Reproduced with permission from Ref [59]. Copyright 2014, Springer Nature. Reproduced with permission from Ref [66]. Copyright 2016, Royal Society of Chemistry. Reproduced with permission from Ref [73]. Copyright 2020, Elsevier. (**b**) Schematic representations of HF etching method (Ti_3_C_2_T_x_ MXene) and (**c**) 2c etching synthesis method (Ti_3_C_2_T_x_ MXene). Reproduced with permission from Ref [61]. Copyright 2016, John Wiley & Sons, Inc. Reproduced with permission from Ref [50]. Copyright 2022, American Chemical Society. (**d**) Schematic illustrations of the molten fluorine salt etching synthesis method (Ti_4_N_3_T_x_ MXene) and (**e**) the organic etching method (Ti_3_C_2_T_x_ MXene). Reproduced with permission from Ref [66]. Copyright 2016, Royal Society of Chemistry. Reproduced with permission from Ref [73]. Copyright 2020, Elsevier

Figure 2a presents a chronological overview of key milestones in conventional MXene synthesis. It highlights the major milestones in the evolution of MXene synthesis methods. In the early stages, fluorine-containing compounds were the dominant etchants. These methods were primarily based on wet-chemical etching (particularly using HF) because it effectively removes the “A” layers from MAX phase precursors. A representative example is the exfoliation of the first MXene (Ti_3_C_2_T_*x*_), which was accomplished by selective etching of monoatomic aluminum layers from a Ti_3_AlC_2_ MAX precursor *via* wet chemical HF etching [39, 48, 49]. Their formation process can be described with following simplified reactions:

(1)

$${\rm{T}}{{\rm{i}}_{\rm{3}}}{\rm{Al}}{{\rm{C}}_{\rm{2}}}\,{\rm{ + }}\,{\rm{3HF}} \to {\rm{Al}}{{\rm{F}}_{\rm{3}}}{\rm{ + 1}}{\rm{.5}}{{\rm{H}}_{\rm{2}}}{\rm{ + T}}{{\rm{i}}_{\rm{3}}}{{\rm{C}}_{\rm{2}}}$$

(2)

$$\eqalign{& {\rm{T}}{{\rm{i}}_{\rm{3}}}{{\rm{C}}_{\rm{2}}}\,{\rm{ + }}\,{\rm{2}}{{\rm{H}}_{\rm{2}}}{\rm{O}} \to {\rm{T}}{{\rm{i}}_{\rm{3}}}{{\rm{C}}_{\rm{2}}}{{\rm{(OH)}}_{\rm{2}}}\,{\rm{ + }}\,{{\rm{H}}_{\rm{2}}}\,{\rm{or}} \cr & {\rm{T}}{{\rm{i}}_{\rm{3}}}{{\rm{C}}_{\rm{2}}}\,{\rm{ + }}\,{\rm{2HF}} \to {\rm{T}}{{\rm{i}}_{\rm{3}}}{{\rm{C}}_{\rm{2}}}{{\rm{F}}_{\rm{2}}}\,{\rm{ + }}\,{{\rm{H}}_{\rm{2}}} \cr} $$

Figure 2b schematically illustrates the HF etching of Ti_3_AlC_2_ MAX phases into Ti_3_C_2_T_x_ MXene [50]. This process involves the following distinct reaction stages: (1) initial grain boundary etching, (2) stepwise interspacing etching, and (3) layer opening. The etching method typically results in a surface termination comprising a mixture of hydroxyl groups (from water) and fluorine groups (from HF) [47, 48]. In addition, the quality of MXenes can be tuned by adjusting parameters such as the HF concentration, reaction temperature, and etching duration [51]. This approach has also been extended to various transition-metal-based MXenes and in-plane/out-of-plane structures, such as Mo_2_Ti_2_C_3_ and Ti_3_MoSiB_2_ [52, 53]. However, a critical drawback was that multilayered MXenes could not be readily exfoliated into single-layer flakes. To address this, in 2013, the Gogotsi group reported that each layer could be expanded more effectively by intercalating organic molecules, specifically using dimethyl sulfoxide (DMSO), which facilitates delamination and improves the dispersion of MXene nanosheets [54]. 

#### Fluorine salt-based etching method

The HF etching method is particularly advantageous because of its straightforward operation at relatively low reaction temperature. However, the use of highly concentrated HF (~ 50 wt%) for etching requires meticulous handling owing to its extreme toxicity and generally results in defect formation (Ti/C vacancy or thin carbon layer), making post-treatment process essential [55–57]. Alternative etching methods have thus been developed to overcome the disadvantages of the direct use of HF. In 2014, the Barsoum group proposed a route to generating HF in-situ during the synthesis process. Using a mixture of HCl and LiF (HCl + LiF etching), Ti_3_C_2_T_x_ MXenes could be synthesized through the etching mechanism of following reactions (Fig. 2c) [58, 59]:

1. $${\rm{LiF + HCl}} \to {\rm{HF + LiCl}}$$
2. $${\rm{T}}{{\rm{i}}_{\rm{3}}}{\rm{Al}}{{\rm{C}}_{\rm{2}}}\,{\rm{ + }}\,{\rm{3HF}} \to {\rm{T}}{{\rm{i}}_{\rm{3}}}{{\rm{C}}_{\rm{2}}}\,{\rm{ + }}\,{\rm{Al}}{{\rm{F}}_{\rm{3}}}\,{\rm{ + }}\,{\rm{1}}.{\rm{5}}{{\rm{H}}_{\rm{2}}}( \uparrow )$$

This approach reduced direct exposure to hazardous HF while still enabling effective etching of the “A” layer. In this in-situ HF-forming condition, the spontaneous intercalation of metal ions (Li^+^) weakens the van der Waals forces between MXene nanosheets. This, in turn, results in increased interlayer spacing and eliminates the need for additional delamination steps (e.g., ultrasonication and manual shaking) [60, 61]. Moreover, this method produces fewer defects, thereby improving the structural integrity of MXenes [62, 63]. Following the development of HCl + LiF etching, subsequent efforts were made to facilitate the expansion of the interlayer spacing between MXene sheets, thereby promoting the delamination process. The amine-assisted delamination process exploited the intercalation of organic molecules to effectively produce Nb_2_C MXene [64, 65]. Molten fluoride salts (KF, NaF, and LiF) were further explored as alternative etchants combining with weaker acids such as H_2_SO_4_ (i.e., molten F-salt etching) [66], as shown in Fig. 2d [67, 68]. In these synthesis steps, metal cations (K^+^, Na^+^, and Li^+^) are intercalated between MXene layers, increasing the interlayer spacing of the nanosheets [69, 70]. A significant advantage of this method is that the varied ionic radii of cations can yield different interlayer spacings. This, in turn, facilitates the delamination of the MXene layers.

Despite these advantages, the in-situ generated HF methods have several drawbacks. They are ineffective at etching grain boundaries, often culminating in incomplete etching of MAX phases in polycrystalline grains [71]. Moreover, it requires multistep delamination processes and complex separation procedures, which substantially obstructs large-scale production [69]. Furthermore, residual HF etchants from fluoride-based reagents pose considerable safety and environmental concerns, further limiting their scalability.

#### Organic solvent-assisted HF etching method

HF etching is commonly conducted in a water-based solution, however, MXenes are generally vulnerable to oxidation in the presence of water, thereby limiting their utilization in water-sensitive applications [72]. In this respect, water-free etching approach was sought after, exploiting polar organic solvents to replace water. Natu et al. synthesized F-terminated Ti_3_C_2_T_x_ using the NH_4_HF_2_ polar organic solvent, as schematically illustrated in Fig. 2e [73]. During etching in organic solvents, the interlayer space was occupied by NH_4_^+^ cation complexes associated with the organic solvent molecules [74, 75]. The intercalation of these organic molecules and/or cation complexes could further weaken the interlayer interactions, leading to a significantly higher yield [70]. 

Organic solvent etching provides advantages over conventional water-based synthesis methods, because MXenes dispersed in organic solvents are less vulnerable to undesirable oxidation than those dispersed in aqueous environments. This enhances their structural integrity [76, 77]. Additionally, the incorporation of organic molecules or ions into the interlayer spaces weakens the interlayer interactions and improves ion transportation [78]. Nevertheless, the use of organic solvents may increase the overall cost and complexity of the synthesis process compared with conventional methods [79]. Moreover, certain organic solvents also pose environmental and safety concerns [80], which present additional challenges for scaling up industrial production. Therefore, a synthesis approach that is best suited to the specific application should be chosen, taking into account of the inherent advantages and drawbacks of each HF-based method.

### Green synthesis method

**Fig. 3:**
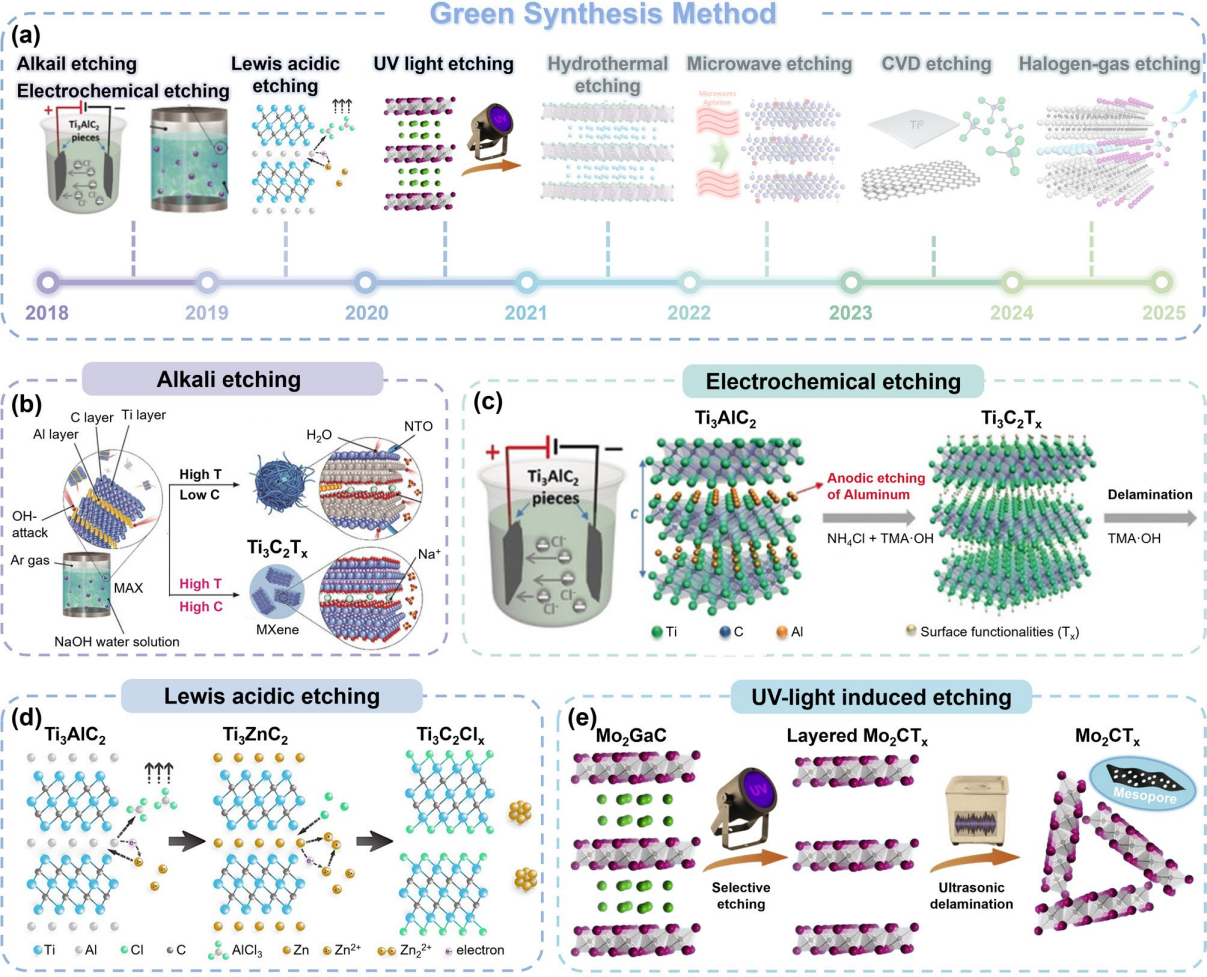
(**a**) Timeline illustrating the progress of Green Synthesis Methods for MXene development, highlighting key advancements in synthesis techniques over time. Reproduced with permission from Ref [82, 107]. Copyright 2018, 2021, John Wiley & Sons, Inc. Reproduced with permission from Ref [89]. Copyright 2017, Royal Society of Chemistry. Reproduced with permission from Ref [94]. Copyright 2019, American Chemical Society. Reproduced with permission from Ref [103, 111, 117]. Copyright 2020, 2022, 2024, Elsevier. Reproduced with permission from Ref [115]. Copyright 2023, The American Association for the Advancement of Science. (**b**) Schematic representation of the alkali etching method (Ti_3_C_2_T_x_ MXene), and (**c**) the method (Ti_3_C_2_T_x_ MXene). Reproduced with permission from Ref [82]. Copyright 2018, John Wiley & Sons, Inc. Reproduced with permission from Ref [89]. Copyright 2017, Royal Society of Chemistry. (**d**) Schematic illustrations of the Lewis acidic etching method with CuCl_2_ salt (Ti_3_C_2_Cl_x_ MXene). Reproduced with permission from Ref [94]. Copyright 2019, American Chemical Society. (**e**) The UV-induced etching method (Mo_2_CT_x_ MXene). Reproduced with permission from Ref [103]. Copyright 2020, Elsevier

**Fig. 4:**
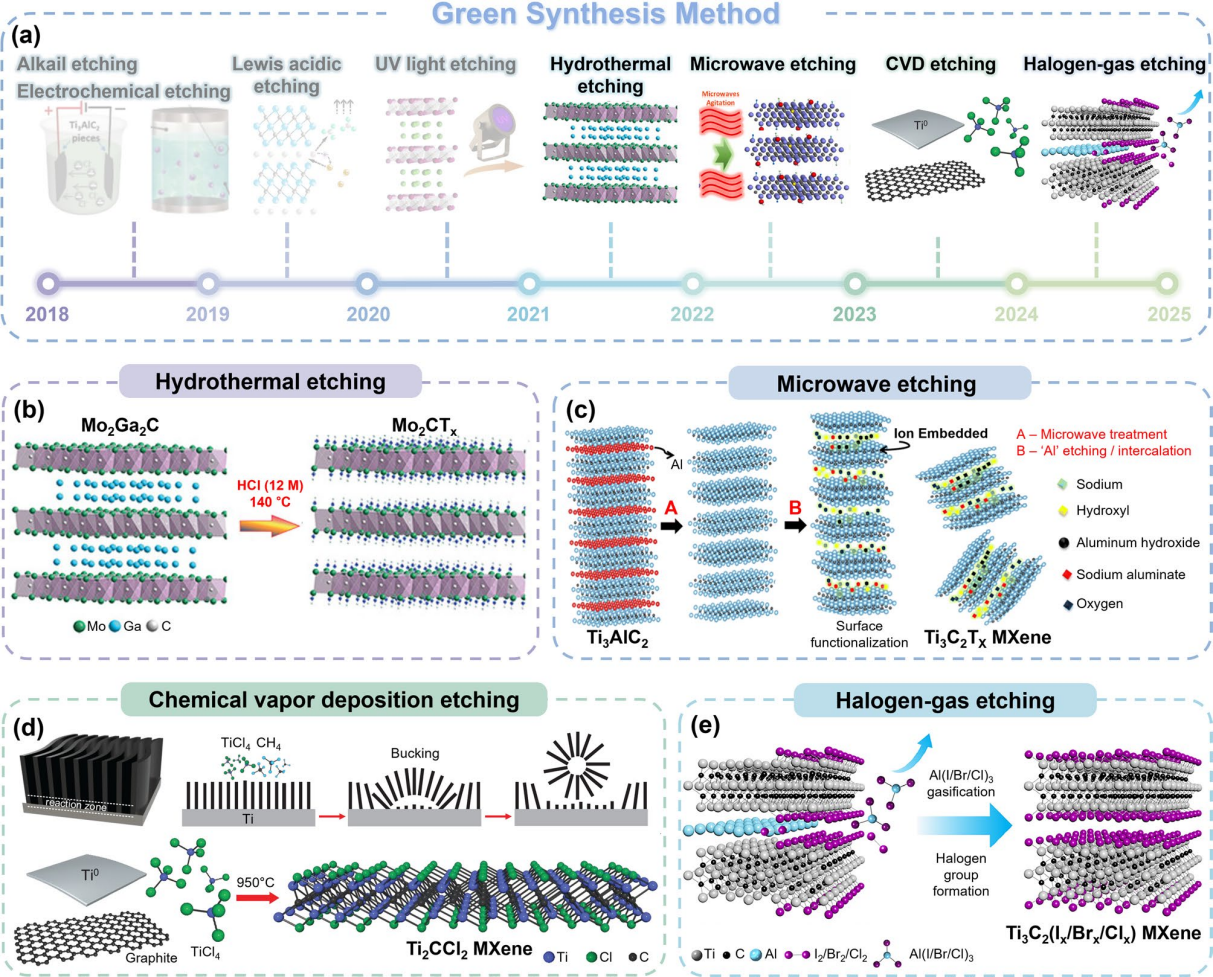
(**a**) Timeline illustrating the progress of Green Synthesis Methods for MXene development, highlighting key advancements in synthesis techniques over time. Reproduced with permission from Ref [82, 107]. Copyright 2018, 2021, John Wiley & Sons, Inc. Reproduced with permission from Ref [89]. Copyright 2017, Royal Society of Chemistry. Reproduced with permission from Ref [94]. Copyright 2019, American Chemical Society. Reproduced with permission from Ref [103, 111, 117]. Copyright 2020, 2022, 2024, Elsevier. Reproduced with permission from Ref [115]. Copyright 2023, The American Association for the Advancement of Science. Schematic representations of (**b**) the etching method with HCl (Mo_2_CT_x_ MXene), and (**c**) the microwave etching method (Ti_3_C_2_T_x_ MXene). Reproduced with permission from Ref [107]. Copyright 2021, John Wiley & Sons, Inc. Reproduced with permission from Ref [111]. Copyright 2022, Elsevier. Schematic illustrations of (**d**) the chemical vapor deposition (CVD) etching method (Ti_2_CCl_2_ MXene) and (**e**) the halogen gas etching method (Ti_3_C_2_T_x_ MXene). Reproduced with permission from Ref [115]. Copyright 2023, The American Association for the Advancement of Science. Reproduced with permission from Ref [117]. Copyright 2024, Elsevier

The development of HF-free MXenes (hereafter referred to as “green MXenes”) has accelerated since the late 2010s in response to the demand for environmentally sustainable synthesis. A timeline of the historical development of green MXenes is illustrated schematically in Fig. 3a (pre-2021, early phase) and Fig. 4a (post-2021, later phase). These include alkali, electrochemical, Lewis acid, UV light-induced, hydrothermal, microwave, chemical vapor deposition (CVD), and halogen gas etching. These methods provide safer and more sustainable pathways for the green synthesis of MXenes, as detailed in this section.

#### Alkali etching method

The selective removal of “A” elements from MAX phases is induced by the high chemical reactivity of M–A bonds compared with M–X bonds. For example, halogen-based etching methods utilize F⁻ and Cl⁻ ions to remove Al as AF_3_ and ACl_3_, respectively [81]. Building upon these prior understandings, the Li group reported that NaOH plays a role similar to that of HF, enabling the etching process (Fig. 3b) [82]. The etching mechanism is described with following reactions:

1. $${\rm{T}}{{\rm{i}}_{\rm{3}}}{\rm{Al}}{{\rm{C}}_{\rm{2}}}\,{\rm{ + }}\,{\rm{NaOH}}\,{\rm{ + }}\,{{\rm{H}}_{\rm{2}}}{\rm{O}} \to {\rm{T}}{{\rm{i}}_{\rm{3}}}{{\rm{C}}_{\rm{2}}}\,{\rm{ + }}\,{\rm{NaAl}}{{\rm{O}}_{\rm{2}}}\,{\rm{ + }}\,{\rm{1}}{\rm{.5}}{{\rm{H}}_{\rm{2}}}$$
2. $${\rm{T}}{{\rm{i}}_{\rm{3}}}{{\rm{C}}_{\rm{2}}}\,{\rm{ + }}\,{\rm{2}}{{\rm{H}}_{\rm{2}}}{\rm{O}} \to {\rm{T}}{{\rm{i}}_{\rm{3}}}{{\rm{C}}_{\rm{2}}}{{\rm{(OH)}}_{\rm{2}}}\,{\rm{ + }}\,{{\rm{H}}_{\rm{2}}}$$

This method produces MXene surfaces with–OH and/or–O terminations, which results in high hydrophilicity [83]. Additionally, the insertion of Na⁺ cations increases the interlayer spacing because Na⁺ has a relatively larger ionic radius than Li⁺ (used in the HCl + LiF etching). Alternatively, strong alkalis such as KOH and TMAOH can also function as effective etchants [84]. For example, the intercalation of TMA^+^ helps regulate the reaction between the Al layer and TMAOH, resulting in a higher yield and finer delamination [85]. However, alkali etching methods confront commercialization challenges because of their low etching capability compared with strong HF-based acidic etching, which is accompanied with incomplete Al removal. Additionally, the OH⁻ attack generates Al hydroxides (Al(OH)_3_) and their dehydrated forms (AlO(OH)) [86], further obstructing reactions with low overall yields.

#### Electrochemical etching method

An alternative approach that does not require acidic or alkali agents is the electrochemical etching method. It enables the direct removal of “A” elements because the weak M-X bond allows for the selective etching of the “A” layer under an applied electric field [87, 88]. By controlling the etching potential and time, “A” sites can be electrochemically etched selectively. Unlike HF-based etching, this approach circumvents the use of fluoride ions and, thereby, yields cleaner MXenes with only Cl-, O-, and OH-terminal groups [87, 89]. Yang et al. first reported the electrochemical etching method to produce Ti_3_C_2_T_x_ as illustrated in Fig. 3c [87]. The electrochemical etching of Ti_3_AlC_2_ was achieved in a binary aqueous electrolyte [46, 57, 73] containing ammonium chloride (NH_4_Cl) and tetramethylammonium hydroxide (TMA⋅OH). The free Cl^−^ from NH_4_Cl electrolyte was oxidated, which formed the -Cl surface termination on the MAX surface [88]. The proposed mechanism for the etching process is as follows:

1. $${\rm{T}}{{\rm{i}}_{\rm{3}}}{\rm{Al}}{{\rm{C}}_{\rm{2}}}\,{\rm{ - }}\,{\rm{3}}{{\rm{e}}^{\rm{ - }}}\,{\rm{ + }}\,{\rm{3C}}{{\rm{l}}^{\rm{ - }}} \to {\rm{T}}{{\rm{i}}_{\rm{3}}}{{\rm{C}}_{\rm{2}}}\,{\rm{ + }}\,{\rm{AlC}}{{\rm{l}}_{\rm{3}}}$$
2. $${\rm{T}}{{\rm{i}}_{\rm{3}}}{{\rm{C}}_{\rm{2}}}\,{\rm{ + }}\,{\rm{2O}}{{\rm{H}}^{\rm{ - }}}\,{\rm{ - }}\,{\rm{2}}{{\rm{e}}^{\rm{ - }}} \to {\rm{T}}{{\rm{i}}_{\rm{3}}}{{\rm{C}}_{\rm{2}}}{({\rm{OH}})_{\rm{2}}}$$

In this approach, the Cl^−^ ion in the electrolyte enables anodic Al etching by breaking the relatively weak Ti − Al bonds. Simultaneously, the intercalation of NH_4_OH opens the edges of the etched anode and promotes further etching of the underlying surfaces. Consequently, the Ti_3_AlC_2_ anode is exfoliated into single layers with–Cl,–O, and–OH terminations. An advanced version of molten-salt-assisted electrochemical etching was subsequently developed by Simon et al. [88]. They reacted carbon sources (CNT and rGO) with Ti and Al powders to prepare Ti_2_AlC MAX. It was then converted into Ti_2_CT_x_ MXene through in-situ electrochemical etching.

These electrochemical etching strategies eliminate the need for acids or bases, enabling a straightforward customization of the surface terminations by selecting appropriate electrolyte compositions [90]. This method is also relatively time-efficient, thereby allowing for rapid synthesis [91]. It typically achieves speeds up to 10 times faster than that of HF etching [92]. By circumventing hazardous etching conditions, it provides a green, economical, and sustainable approach. Nevertheless, it generally requires additional post-treatment with acid to achieve complete removal of the “A” layers [92]. Moreover, precise control over the applied voltage is necessary to prevent over-etching and/or undesired defects. These challenges necessitate further optimization to enhance their practicality.

#### Lewis acidic etching method

As an alternative green chemical approach, Lewis acidic molten salts are viable substitutes for conventional etching methods. This method selectively removes the “A” layers in a molten salt liquid medium, without the need for an acid-based solvent [93]. Molten salts, forming typically above 500 ℃, eliminate–O and–OH functional groups, thus allow for controlled halogenated-terminations (–Cl,–Br, and–I). For example, the Huang group synthesized Ti_3_C_2_Cl_2_ MXene using ZnCl_2_ at 550 ℃, as depicted in Fig. 3d [94]. The overall process is described by the following reactions:

1. $${\rm{T}}{{\rm{i}}_{\rm{3}}}{\rm{Al}}{{\rm{C}}_{\rm{2}}}{\rm{ + 1}}{\rm{.5ZnC}}{{\rm{l}}_{\rm{2}}} \to {\rm{T}}{{\rm{i}}_{\rm{3}}}{\rm{Zn}}{{\rm{C}}_{\rm{2}}}{\rm{ + 1}}{\rm{.5Zn + AlC}}{{\rm{l}}_{\rm{3}}} \uparrow $$
2. $${\rm{T}}{{\rm{i}}_{\rm{3}}}{\rm{Zn}}{{\rm{C}}_{\rm{2}}}{\rm{ + 1}}{\rm{.5ZnC}}{{\rm{l}}_{\rm{2}}} \to {\rm{T}}{{\rm{i}}_{\rm{3}}}{{\rm{C}}_{\rm{2}}}{\rm{ + 1}}{\rm{.5Zn + AlC}}{{\rm{l}}_{\rm{3}}} \uparrow $$
3. $${\rm{T}}{{\rm{i}}_{\rm{3}}}{{\rm{C}}_{\rm{2}}}{\rm{ + ZnC}}{{\rm{l}}_{\rm{2}}} \to {\rm{T}}{{\rm{i}}_{\rm{3}}}{{\rm{C}}_{\rm{2}}}{\rm{C}}{{\rm{l}}_{\rm{2}}}{\rm{ + Zn}}$$

The mechanism involves the Zn^2+^ ions in molten ZnCl_2_, which function as Lewis acids by accepting electrons. Owing to the weakened bonding between Al and Ti atoms, Al is oxidized to Al^3+^ *via* a redox process. The resulting Al^3+^ ion then combines with Cl^−^ to form AlCl_3_, which (owing to its low boiling point; ~ 180 ℃) vaporize at 550 ℃. This drives the reaction forward and ensures efficient etching. Based on these observations, the synthesis of MXenes was expanded further by modifying various A-site elements (Zn, Al, Si, and Ga) in the MAX phases and optimizing the Lewis acid melt composition [95, 96]. An example is the Ti_3_SiC_2_ as a non-Al MAX phase. It was synthesized in molten CuCl_2_ at 750 ℃ using ammonium persulfate (APS) solution to remove residual Cu particles [97]. Additionally, a series of Ti-, Nb-, and Mo-based MAX phases were exfoliated successfully using various chloride-based molten salts, demonstrating the versatility of this approach [98–100]. Although the Lewis acid method provides a broader etching range and improved chemical safety, a primary limitation is the residual metallic impurities remaining after the etching process. Therefore, additional acid treatment for complete removal is necessary [101, 102]. Also, etching in molten salts requires temperatures up to ~ 800 °C and extended dwell times (~ 24 h), which can lead to higher energy consumption compared to wet etching methods.

#### Ultraviolet (UV)-induced etching method

To overcome the high-temperature limitations of molten salt-based etching, recent research has focused on photochemical strategies that enable MXene synthesis at ambient or near-room temperature conditions. One such advancement was reported by Mei et al., as demonstrated in Fig. 3e [103]. In this method, an ultraviolet (UV)-induced selective etching was carried out to synthesize mesoporous Mo_2_C MXenes from Mo_2_Ga_2_C MAX using phosphoric acid as the etchant. Since Mo_2_Ga_2_C is a strong UV-absorbing material, it facilitates the removal of Ga layers by accelerating the surface reactions under UV irradiation, which weakened the Ga bonds and promoted selective etching [104]. UV irradiation helps induce the “A” layer extraction without requiring high temperatures, making the process more environmentally friendly. Significantly, UV irradiation has been demonstrated to be a preferable method because it eliminates the need for hazardous and highly corrosive acids. In addition, this approach enables green MXene synthesis within a few hours, thereby reducing the processing time compared with conventional HF methods. However, the applicability of this method is limited if the MAX candidates are sensitive to UV light. Additionally, the UV light interacts with the TiO_2_ causing O∙ and OH∙ radical formation, which results in further oxidation. Therefore, further investigation is required to achieve high-quality green MXenes under these UV conditions.

#### Hydrothermal etching method

The hydrothermal etching method utilizes high-temperature and high-pressure conditions that provide a sufficient driving force to selectively remove “A” elements from MAX phases (Fig. 4a displaying the later phase (post-2021)) [105, 106]. Song et al.. reported the synthesis of Mo_2_CT_x_ MXenes using an HCl-based hydrothermal approach to selectively remove Ga from Mo_2_Ga_2_C (Fig. 4b) [107]. The strong interaction between OH⁻/Cl⁻ ions and the Ga elements enabled an effective etching of MAX phases. During the process, the extraction of Ga expanded the grain boundaries, which promoted Cl⁻ ion penetration and further weakened the Mo-Ga bonds, facilitating efficient layer separation. To achieve higher MXene yields, Yang et al. further developed a hydrothermally assisted intercalation approach using tetramethylammonium hydroxide (TMAOH) [108]. The Al(OH)_4_^–^, formed from the reaction between TMAOH and Al, adhered to the Ti-layer and caused further expansion of the interlayer spacing. Despite its advantages, hydrothermal etching requires specialized autoclave systems capable of withstanding high temperatures and pressures, increasing equipment costs [109, 110]. Furthermore, impurities such as residual aluminum hydroxides (Al(OH)₃) and their dehydrated forms (AlO(OH)) are frequently produced during the etching process, hindering the complete reaction. These impurities decrease the overall yield and necessitate additional post-treatment steps (e.g., acid washing) to obtain high-purity green MXenes.

#### Emerging alternative etching methods

Recently, other advanced techniques have been proposed for green MXene synthesis. For instance, Mahabari et al. reported the NaOH-based microwave-assisted synthesis of MXenes, as illustrated in Fig. 4c [111]. Under microwave radiation, the interlayer water evaporates rapidly, enabling the synthesis of larger lateral-sized few-layer MXenes in 30 min (significantly faster than the typical HF etching procedures). This microwave-assisted technique provided uniform heating, homogeneous nucleation, and rapid crystal development, yielding MXene crystallites of finite sizes, in conjunction with advantages such as cost-effectiveness and convenience of operation [112]. Nevertheless, the low synthesis yield (< 10%) remains a major constraint [113], and localized overheating from microwave exposure may degrade the quality of MXene [114]. 

Most synthesis techniques inherently contain surface terminations, and this significantly hinders the production of termination-free MXenes (“clean MXenes”). CVD (Chemical Vapor Deposition) provides a unique advantage over other alternatives because it enables MXene fabrication through a bottom-up process. Talapin et al. reported that CVD growth could be used to directly synthesize Ti_2_CCl_2_ MXenes without requiring a parent MAX phase (Fig. 4d) [115]. Under a high-temperature condition, the reaction between Ti, graphite, and TiCl_4_ facilitates the deposition of Ti₂CCl₂ MXene carpets with complex spherulite-like morphologies. The versatility of this approach was attributed to its dependence on the growth temperature, gas pressure, gas flow rate, catalyst type, and cooling procedure [116]. Although CVD requires expensive processing conditions and high energy that may limit its industrial feasibility, it is significant because it represents a fundamentally different approach capable of producing “clean MXenes” without inherent surface terminations.

A solvent-free gas-phase etching method is another appropriate option for synthesizing green MXenes with precise control over the surface terminations. The gas-phase reactor can selectively etch “A” elements while simultaneously incorporating gas-induced functional groups (–I_x_,–Br_x_, and–Cl_x_). Recently, Zhu et al. introduced a solvent-free gas-phase approach using halogen and hydrogen halide gases (Cl_2_, Br_2_, I_2_, HCl, HBr, and HI), as illustrated in Fig. 4e [117]. Compared with conventional wet chemical etching, where the etchant diffusion is limited by ionic interactions, gas-phase etching utilizes higher kinetic mobility of gaseous reactants, ensuring uniform etching. A key advantage of this method is that it eliminates the need for extensive purification steps because the gaseous by-products from the etching process can be easily separated from the final MXene product. This method is cost-effective and straightforward, which makes it suitable for large-scale production. However, it is important to note that the management and handling of reactive or toxic gases require stringent safety measures.

## Green MXenes for battery applications

As reviewed in previous sections, the physical, chemical, and structural properties of MXenes can vary depending on the synthesis strategy employed. Consequently, their application in battery systems exhibits diverse characteristics. Considering the wide range of battery technologies, including the current LIBs and future systems such as Li metal batteries and Li-sulfur batteries, we overview the applications of green MXenes within each specific system.

### Green MXene for Li-ion batteries (LIBs)

#### Utilization of surface terminations

*Over the past decade*,* MXenes have been investigated extensively for LIB applications*. To systematically comprehend these applications, we classify them into two main groups based on the strategies employed: 4.1.1 Utilization of surface terminations and 4.1.2 Structural engineering (porous and 3D architectures). Surface terminations directly influence electrochemical behavior by modifying the charge transport and ion diffusion kinetics. Structural engineering enhances electrode stability and maximizes the accessible surface area for improved capacity retention. In this section, we focus on the role of surface termination in improving the electrochemical performances of MXenes in LIBs.

**Fig. 5:**
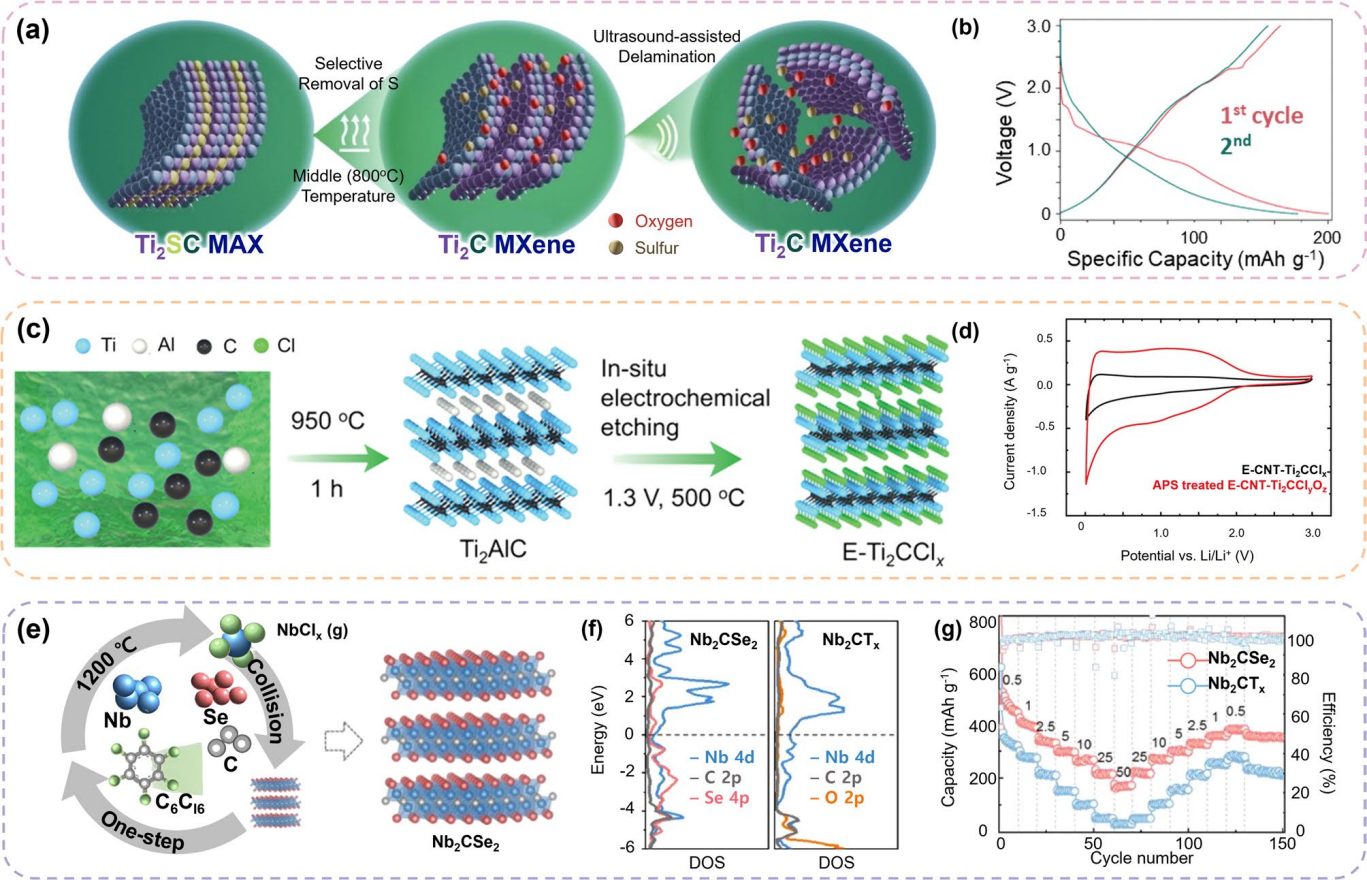
Examples of Green MXene applications in Li-ion batteries (LIBs). (**a**) Schematic representation of the thermal reduction process used to synthesize Ti_2_C MXene and (**b**) electrochemical characterization of discharge/charging plot of Ti_2_C MXene. Reproduced with permission from Ref [120]. Copyright 2020, Elsevier. (**c**) Schematic illustration of the fabrication of E-Ti_2_CCl_x_ and (**d**) cyclic voltammetry (CV) cycle of E-CNT-Ti_2_CCl_x_ and APS-treated E-CNT-Ti_2_CCl_y_O_z_. Reproduced with permission from Ref [88]. Copyright 2022, John Wiley & Sons, Inc. (**e**) Schematic representation of one-step preparation process of Nb2CSe2 (**f**) DOS of Nb_2_CSe_2_ and Nb_2_CT_x_. (**g**) rate capability of Nb_2_CSe_2_ and Nb_2_CT_x_. Reproduced with permission from Ref [122]. Copyright 2023, Elsevier

The primary advantage of MXenes is their capability to selectively contain specific surface functional groups such as–F,–O,–OH, and–Cl. In the green MXene synthesis approach, the elimination of–F functional groups was often attempted because they were suspected to limit Li^+^ storage by reducing the ion diffusion kinetics [118]. Meanwhile, oxygen termination (–O) was believed to enhance Li intercalation with lower Li^+^ migration barrier (~ 0.2 eV) [119] In this regard, Mei et al. synthesized O-terminated Ti_2_C MXenes *via* thermal reduction, as depicted in Fig. 5a [120]. The O-terminated Ti_2_C MXene exhibited a high initial capacity of 200 mA g^− 1^ and a capacity retention of 88% (Fig. 5b). The high capacity was attributed to the delaminated MXene layers, which enhanced the active surface area by increasing the surface exposure. Liu et al.. reported an E-CNT-Ti_2_CCl_y_O_z_ *via in-situ* electrochemical etching [88]. Initially, the surface functional groups on the MXene were uniformly terminated by Cl (Fig. 5c). After washing with ammonium persulfate (APS), most of the–Cl groups were replaced by–O groups. The presence of–O terminations was claimed to facilitate rapid Li^+^ diffusion owing to a reduced migration barrier, while maintaining a moderate adsorption energy (~ -0.90 eV) that ensured stable Li^+^ retention (Fig. 5d) [121]. This balanced interplay between ion mobility and surface interactions enhanced the overall Li storage capacity of the green MXene.

MXenes, terminated with strongly electronegative groups such as–O,–OH, and–F, typically exhibit low electronic conductivities due to localized electrons, which adversely affect the charge transport in the electrode. To overcome this issue, Pang et al.. synthesized Se-terminated niobium carbide MXene (Nb_2_CSe_2_) using a one-step vapor activation method (Fig. 5e) [122]. This bottom-up strategy enabled the direct formation of a layered Nb_2_CSe_2_ structure from a stoichiometric mixture of Nb, C, Se, and C_6_Cl_6_ without acid etching. During synthesis, C_6_Cl_6_ decomposes to form halogen-containing gases that react with Nb to generate volatile NbCl_x_ species. These then react with Se and C to form a layered Nb_2_CSe_2_ structure *via* a gas-solid reaction without requiring a MAX-phase precursor. Compared with O-terminated MXenes,–Se termination significantly enhanced the delocalized electron availability, as evidenced by DFT calculations that revealed an increased density of states at the Fermi level. It was contributed by both Nb 4d and Se 4p orbitals (Fig. 5f). This modification led to an increase in conductivity (~ 7.14 × 10^4^ S m^− 1^; over 2,000 times higher than that of Nb_2_CT_x_) and enhanced electrochemical performance even at 50 C rate (Fig. 5g).

#### Structural engineering: porous and 3D architecture

**Fig. 6:**
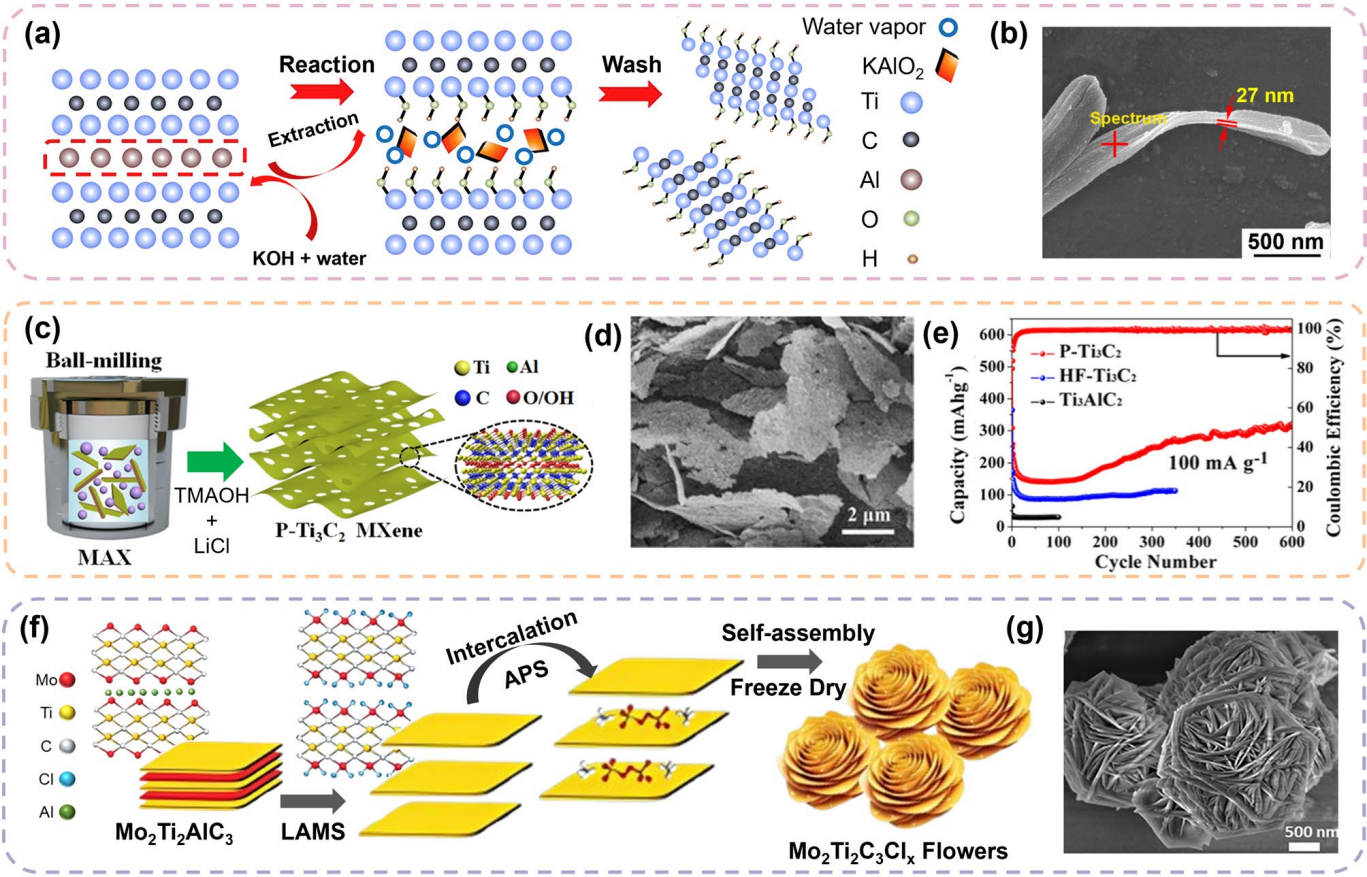
Green MXene synthesis strategies for structural engineering applications. (**a**) Schematic illustration of the synthesis process of Ti_3_C_2_(OH)_2_NRs, (**b**) SEM image of Ti_3_C_2_(OH)_2_NRs. Reproduced with permission from Ref [123]. Copyright 2019, Elsevier. (**c**) Schematic representation of the preparation of p-Ti_3_C_2_ by ball milling method and the corresponding (**d**) SEM image of p-Ti_3_C_2_ sheets, (**e**) electrochemical performance of pTi_3_C_2_ and HF-Ti_3_C_2_. Reproduced with permission from Ref [124]. Copyright 2020, American Chemical Society. (**f**) Schematic illustration of self-assembled Mo_2_Ti_2_C_3_Cl_x_, with (**g**) SEM image showing the flower-like morphologies. Reproduced with permission from Ref [125]. Copyright 2024, John Wiley & Sons, Inc

Green MXene sheets have been used in LIBs, both in their inherent two-dimensional (2D) form or as alternative three-dimensional (3D) architectures. Zhu et al.. developed a 3D Ti_3_C_2_T_x_ nanoribbon structure using a fluoride-free method based on KOH and water vapor. KOH functioned as a mild etchant to selectively remove Al by forming soluble aluminate complexes, while water vapor generated localized steam pressure that aided mechanical exfoliation along the interlayer planes (Fig. 6a) [123]. This synergistic chemical-physical process efficiently transformed the material into open, ribbon-like architectures that minimized layer restacking. The resulting structure exhibited an elongated, flexible nanoribbon with a curved, layered morphology, as shown in Fig. 6b. This open architecture facilitated enhanced electrolyte infiltration and accelerated Li^+^ transport, exhibiting a reversible capacity of 143.4 mAh g^− 1^ after 250 cycles at 0.1 A g^− 1^. Tao et al. proposed a scalable fluorine-free strategy to synthesize porous Ti_3_C_2_ (p-Ti_3_C_2_) MXene *via* ball milling using tetramethylammonium hydroxide (TMAOH) and LiCl as activating agents (Fig. 6c) [124]. During milling, localized mechanical generates interlayer fractures and surface defects that form a porous framework. Concurrently, TMAOH functions as an effective etchant and selectively removes Al from the MAX phase. Meanwhile, LiCl enhances exfoliation and assists in interlayer charge redistribution and structural transformations. The resulting p-Ti_3_C_2_ exhibited a rough, layered morphology with hierarchical porosity (Fig. 6d) and significantly enhanced surface area (38.93 m^2^ g^− 1^; approximately eight times larger than that of conventional HF-etched Ti_3_C_2_). This porous 3D framework increased the number of accessible active sites and provided efficient pathways for Li^+^ diffusion and electrolyte penetration, significantly outperforming HF-Ti_3_C_2_ (Fig. 6e). Gandla et al. developed a flower-like 3D green MXene [125] (Mo_2_Ti_2_CCl_x_) with engineered in-plane microporosity using a Lewis acid molten salt strategy followed by ammonium persulfate (APS) intercalation and freeze-drying (Fig. 6f). A distinctive feature of this approach is the spontaneous formation of hierarchical morphology through self-assembly. It is induced by the APS-induced interlayer expansion and solvent dynamics during freeze-drying. Specifically, APS weakens the interlayer bonding and facilitates exfoliation, whereas freeze-drying removes the solvent under non-equilibrium conditions. This guides the nanosheets to reorganize into a flower-like 3D structure (Fig. 6g). The resulting morphology exhibited a high surface area (38.0 m^2^ g^− 1^) and interconnected mesopores (2–5 nm), which enhanced electrolyte accessibility and Li^+^ diffusion. When applied as an LIB anode, Mo_2_Ti_2_C_3_Cl_x_-1322 provided a specific capacity of 324 mAh g^− 1^ at 0.2 A g^− 1^ and retained 97% capacity retention over 500 cycles at 0.5 A g^− 1^.

### Green MXenes for Li metal batteries (LMBs)

#### Green MXenes as lithiophilic host

**Fig. 7:**
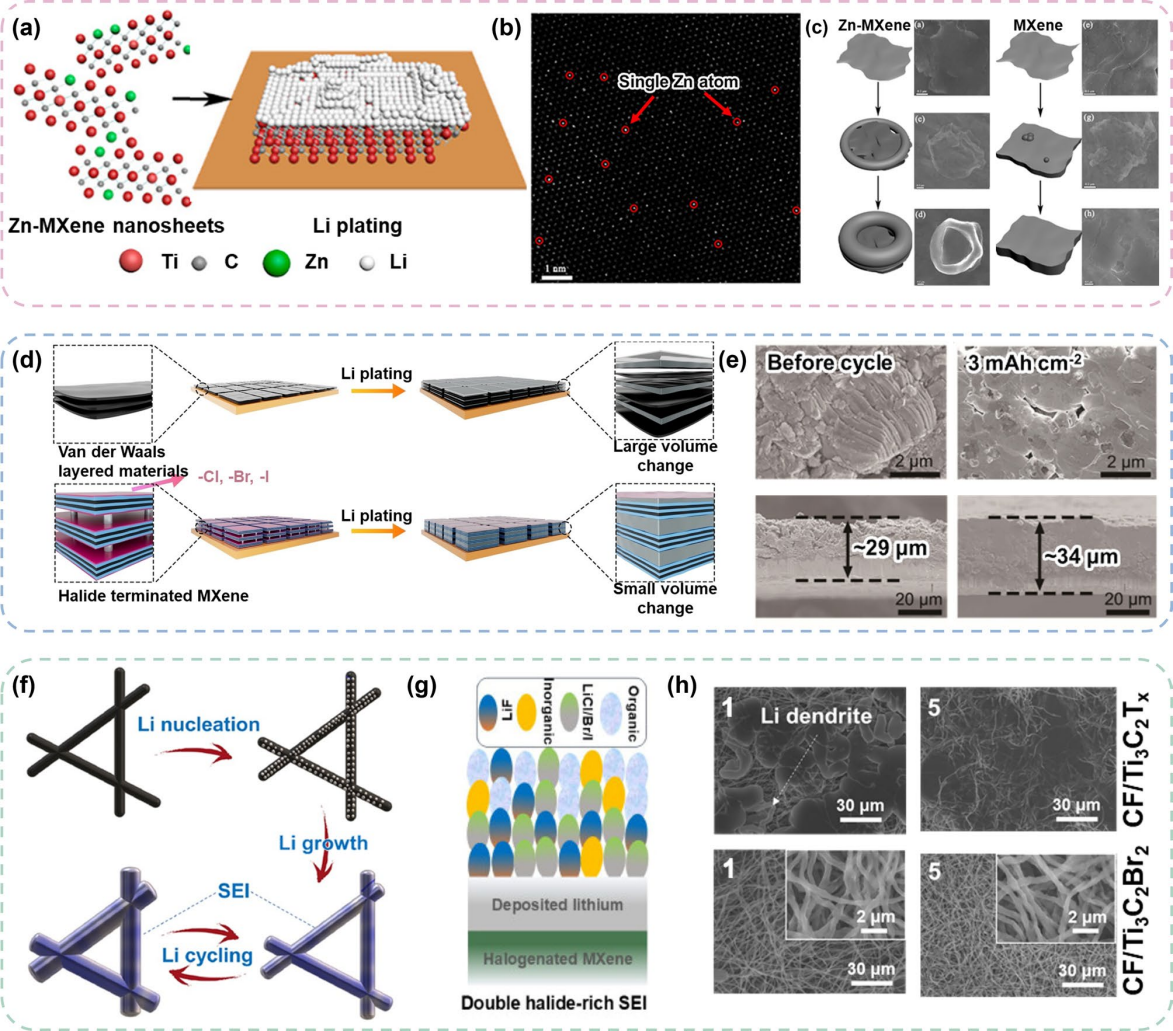
Examples of Green MXene used as lithiophilic host for LMBs. (**a**) Schematic illustration of Zn-MXene nanosheet synthesized via the Lewis acid molten salt method, (**b**) and the corresponding TEM image. (**c**) SEM images comparing the Li plating behaviors of Zn-MXene and conventionally synthesized MXene. Reproduced with permission from Ref. [126]. Copyright 2020, American Chemical Society. (**d**) Schematic representation of Li plating and stripping behaviors on halide-terminated MXene, followed by (**e**) SEM images showing structural variations. Reproduced with permission from Ref. [127]. Copyright 2024, John Wiley & Sons, Inc. (**f**) Schematic illustrations for the Li plating and cycling on the halogenated 3D CF/MXene and (**g**) the formation of a double halogenated SEI layer. (**h**) SEM image comparing CF and 3D CF/MXene hosts after Li plating. Reproduced with permission from Ref. [128]. Copyright 2024, Elsevier.

The high electrical conductivity, abundant polar surface functionals with strong lithiophilicity, and robust structure of green MXenes render these particularly suitable as functional additive for Li metal anodes. These properties enable efficient Li nucleation, stable Li metal deposition, and the suppression of dendrite growth, which are critical challenges in the development of LMBs. Gu et al. fabricated Ti_3_C_2_Cl_x_ MXene incorporating Zn (Zn-MXene) using Lewis acid molten salt method, as shown in Fig. 7a [126]. The embedded Zn atoms in green MXene (Fig. 7b) aided in presenting high lithiophilicity, which facilitated reversible Li nucleation and growth. It was reported that the Zn-MXene host induced unusual Li plating behavior (Fig. 7c), in which a bowl-shaped Li formation promoted the rapid plating of Li metal compared with the conventionally synthesized Ti_3_C_2_T_x_ MXene, suppressing dendrite growth.

Wu et al.. also utilized the Lewis acid molten salt method to produce halide-terminated (− Cl, −Br and −I) MXene, which is specifically designed to adapt to volume variations during Li plating and stripping. In this structure, Li functions as a “zipper”: it reassembles adjacent MXene layers into a MAX-like phase, significantly improving the structural stability (Fig. 7d) [127]. A halide-rich SEI was formed by the electrochemical removal of terminal groups from halogenated MXenes, which ensured enhanced stability and rapid Li^+^ diffusion within the SEI. Owing to these characteristics, the halogenated green MXenes demonstrated minimal volume variations even under fast plating conditions (Fig. 7e). Another example of a host with a halogenated green MXene for Li metal anodes was prepared by non-fluorinated acid etching combined with a polymer backbone [128]. These halogenated MXenes were prepared using the Lewis acid molten salt method and mechanically mixed with polyacrylonitrile (PAN). After mixing, the PAN was carbonized to fabricate a 3D carbon fiber (CF)/MXene framework (Fig. 7f). This 3D framework with green MXenes exhibited high electrochemical stability in a Li symmetric cell owing to the formation of a double halide-rich SEI consisting of LiF, LiCl, LiBr, and LiI (Fig. 7g). Consequently, Ti_3_C_2_Br_2_ MXene effectively suppressed the dendrite growth, whereas conventional Ti_3_C_2_T_x_ was vulnerable to dendrite formation (Fig. 7h).

The unique properties of green MXenes, including uniform surface termination, high electrical conductivity, and robust structural stability, make these highly advantageous as Li metal hosts. Their lithiophilicity can be enhanced further by tuning their surface chemistry through single-atom metal doping and customized termination. This, in turn, facilitates efficient Li nucleation and deposition. Additionally, the lower defect density of the green MXenes provides superior mechanical and chemical stability. Given these advantages, green MXenes have significant potential as Li-metal hosts to contribute to the development of high-performance, long-lasting LMBs.

#### Interfacial engineering of Li metal anodes

**Fig. 8:**
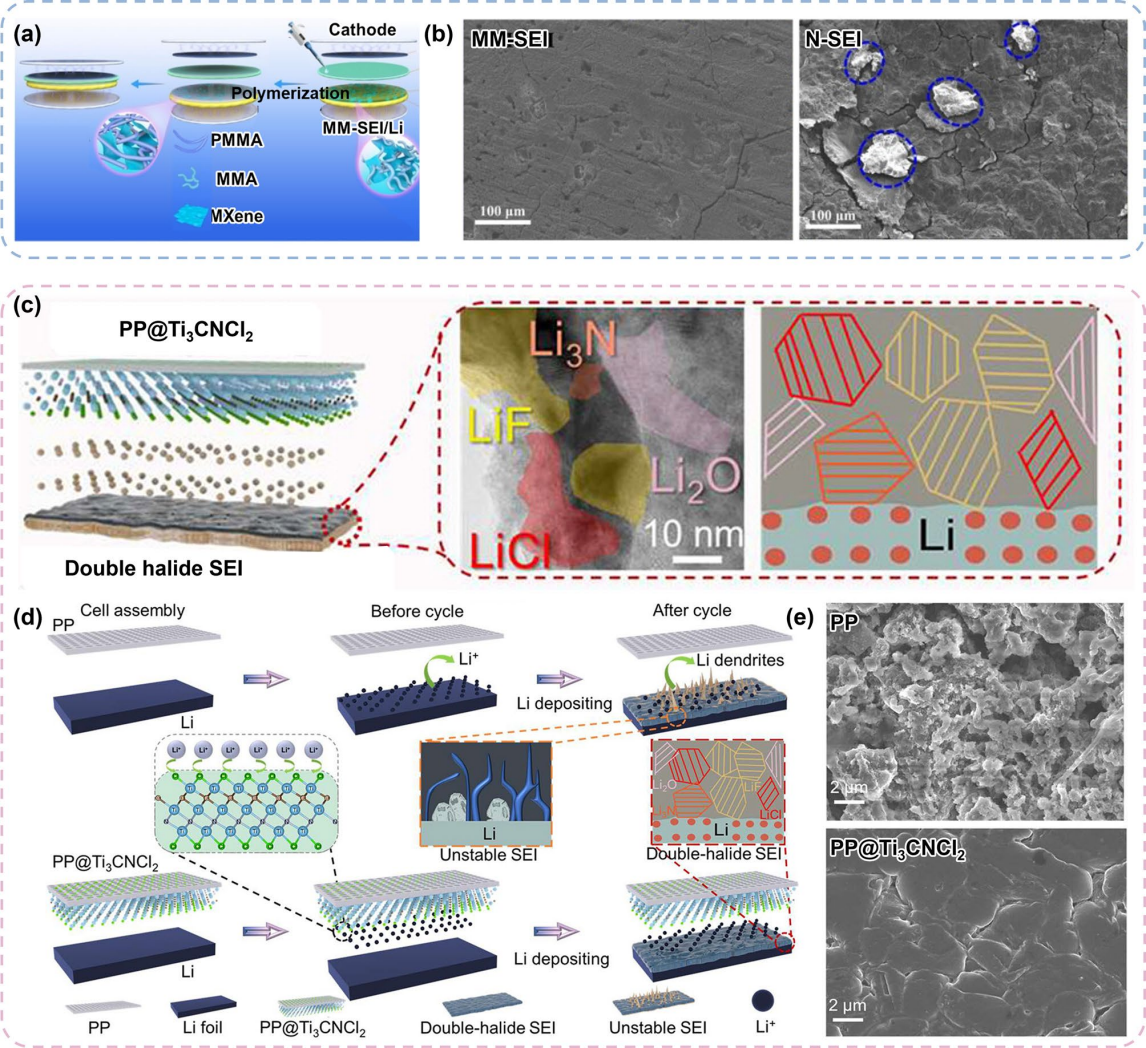
Representative examples for interfacial engineering for Li metal anodes. (**a**) Schematic illustration of Green MXene@PMMA composite as an interface protective layer (MM-SEI) for all-solid-state battery. (**b**) SEM images showing post-cycling surface morphologies with MM-SEI and N-SEI. Reproduced with permission from Ref [129]. Copyright 2022, Elsevier. (**c**) Schematic illustration of PP@Ti_2_CNCl_2_ separator and the formation of a double-halide SEI layer. (**d**) Comparison of Li metal interface between conventional PP and green Ti_3_CNCl_2_ MXene-coated separator. (**e**) SEM images after Li depositions, comparing the PP separator and PP@Ti_3_CNCl_2_ separator. Reproduced with permission from Ref [130]. Copyright 2023, American Chemical Society

The applications of green MXenes have expanded to regulate Li metal interfaces beyond the role of Li metal hosts. More specifically, attempts have been undertaken to use green MXenes as a protective layer for Li metal by leveraging their capability to create a uniform ion flux at the Li metal interface. Huang et al. introduced a green MXene@poly (methyl methacrylate) (PMMA) composite as an interface-protective layer (MM-SEI) for all-solid-state LMB (Fig. 8a) [129]. They mixed methyl methacrylate (MMA) monomers with green MXenes and polymerized MMA on Li metal to form an artificial SEI. With the charge redistribution effect of the green MXene and the high ionic conductivity of PMMA in the MM-SEI, the interface of the Li metal was maintained stably after cycling. The homogeneous surface with unique terminations of the green MXenes outperformed conventional MXenes when these were in contact with the interface of the LMBs (Fig. 8b). Owing to the remarkable Li^+^ diffusion capability of green MXene, the ionic conductivity of the MM-SEI also increased to 1.65 mS cm^− 1^ at room temperature. This high ionic conductivity, combined with its mechanical and chemical stability, enabled a low overpotential and high cyclability.

Modifying the separator in LMB can be an alternative approach to regulating the Li metal interface rather than directly coating a protective layer on Li metal. Zhang et al. reported Ti_3_CNCl_2_ MXene coated onto a polypropylene (PP) separator from a molten salt etchant method (Fig. 8c) [130]. The coated green MXene improved the electrolyte wettability and reduced the ion diffusion barriers, thereby enhancing the Li^+^ diffusion kinetics. More importantly, it contributed to the formation of a robust SEI structure with uniformly distributed LiF and LiCl. It was demonstrated that PP coated with Ti_3_CNCl_2_ MXene forms a stable interface because of the LiCl/LiF double halide SEI created by the Cl functional groups on the surface of green MXene (Fig. 8d). The stable interface promoted faster ion movement and smooth flux of Li^+^ diffusion. Consequently, the use of the PP@Ti_3_CNCl_2_ separator induced a significantly more uniform Li plating compared with the cell with the pristine PP separator (Fig. 8e). Variations in the surface composition significantly affect electrolyte wettability, Li adsorption energy, charge density, SEI components, etc. In this regard, green MXene is expected to be prominent as interface modifier for Li metal anodes because its customized surface terminations may function as catalytic sites for Li deposition and can be tuned depending on the synthesis method used.

### Green MXenes for Li-Sulfur batteries (LSBs)

#### Green MXenes for sulfur cathode host

In the development of LSBs, conducting hosts have been imperative because they compensate for the poor electrical conductivity of sulfur and encapsulate lithium polysulfides (LiPSs) that are produced during cycling of sulfur cathodes. In this regard, various 1D–3D carbon host materials have been evaluated for the formation of sulfur composites [131, 132]. Importantly, a key requirement for a preferable sulfur host is a strong binding energy with LiPSs. This is because it suppresses their dissolution into the electrolyte and mitigates the shuttle effect, which is crucially linked to the cycle life of LSBs. Green MXenes have emerged as potential sulfur host materials that fulfill these requirements, surpassing conventional conducting carbons.

**Fig. 9:**
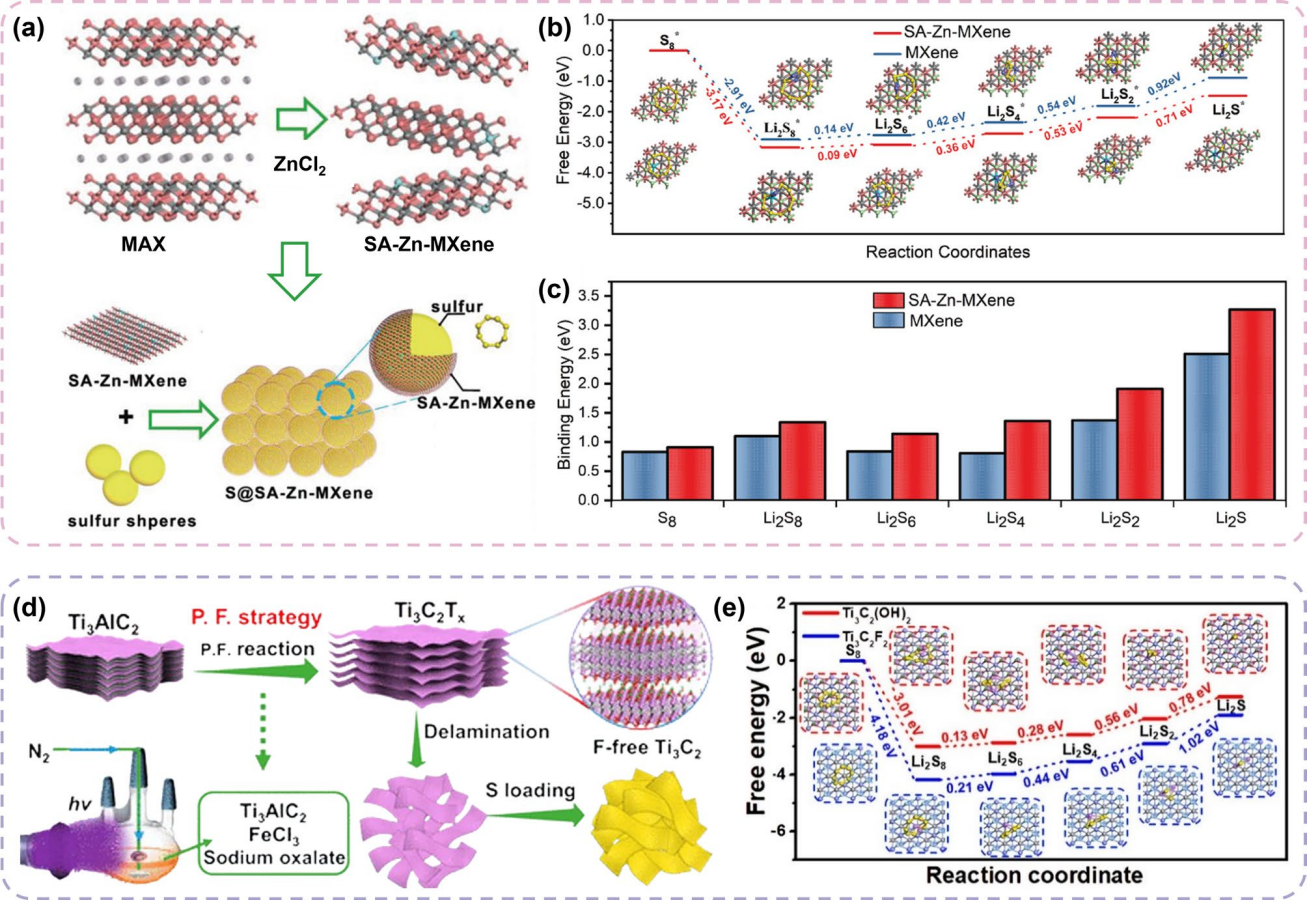
Examples of Green MXenes used as sulfur hosts in LSBs and their advantages over conventional MXene. (**a**) Schematic illustration of the fabrication process of S@SA-Zn-MXene. (**b**) Gibbs free energy comparison and (**c**) binding energy analysis between SA-Zn-MXene and conventional MXene, highlighting the improved polysulfide adsorption capability. Reproduced with permission from Ref [133]. Copyright 2020, John Wiley & Sons, Inc. (**d**) Schematic illustration of the fabrication of S@Ff-Ti_3_C_2_ MXene cathode using the photo-Fenton (P.F.) reaction. (**e**) Comparison of LiPS reaction energy barriers between Ff-Ti_3_C_2_ and conventional MXene. Reproduced with permission from Ref [134]. Copyright 2022, American Chemical Society

Single-atom doped green MXenes have been considered a practical approach to mitigating LiPS shuttling and accelerating the conversion reaction simultaneously. Figure 9a presents an example of utilizing a single-atom catalyst with green MXene as an LSB cathode host. Single-atom Zn-MXene (SA-Zn-MXene) was synthesized using Lewis acid molten salt method with ZnCl_2_ [133]. This composite catalyzedd the conversion reactions of LiPSs by lowering the energy barriers from Li_2_S_4_ to Li_2_S_2_/Li_2_S (Fig. 9b). Additionally, it established strong interactions with LiPSs owing to the high electronegativity of SA-Zn on the green MXene surface (Fig. 9c). It was reported that the uniformly distributed Zn atoms facilitated the nucleation of Li_2_S_2_/Li_2_S on the MXene layers during the redox reactions, whereas the strong LiPS adsorption enhanced the cycle performance. Owing to these effects of the green MXene-based sulfur host, SA/Zn-MXene exhibited a stable cyclability (350 cycles) and high sulfur utilization (5.3 mg cm^− 2^). Xiao et al. developed a photo-Fenton (P.F.) reaction to synthesize fluorine-free Ti_3_C_2_ MXenes (Ff-Ti_3_C_2_) for sulfur host (Fig. 9d) [134]. The continuous production of reactive oxygen in the P.F. reaction species promoted the deintercalation of Al layers while simultaneously forming–OH and–O terminations. The increased charge accumulation on the surface of Ff-Ti_3_C_2_ functioned as an abundant electron reservoir. This also promoted the kinetics of lithiation, strengthened host–guest interactions, and facilitated Li-ion diffusion. Furthermore, its higher binding energy with LiPSs and lower Li_2_S cluster dissociation energy barriers contributed to a stronger LiPS shuttle inhibition and higher catalytic effect of Ff-MXene (Fig. 9e). As a result, S@Ff-MXene exhibited faster reaction kinetics than conventional MXene, demonstrating a higher exchange current density (0.128 mA cm^− 2^) than F-MXene (0.058 mA cm^− 2^).

#### Green MXenes for separator of LSBs

**Fig. 10:**
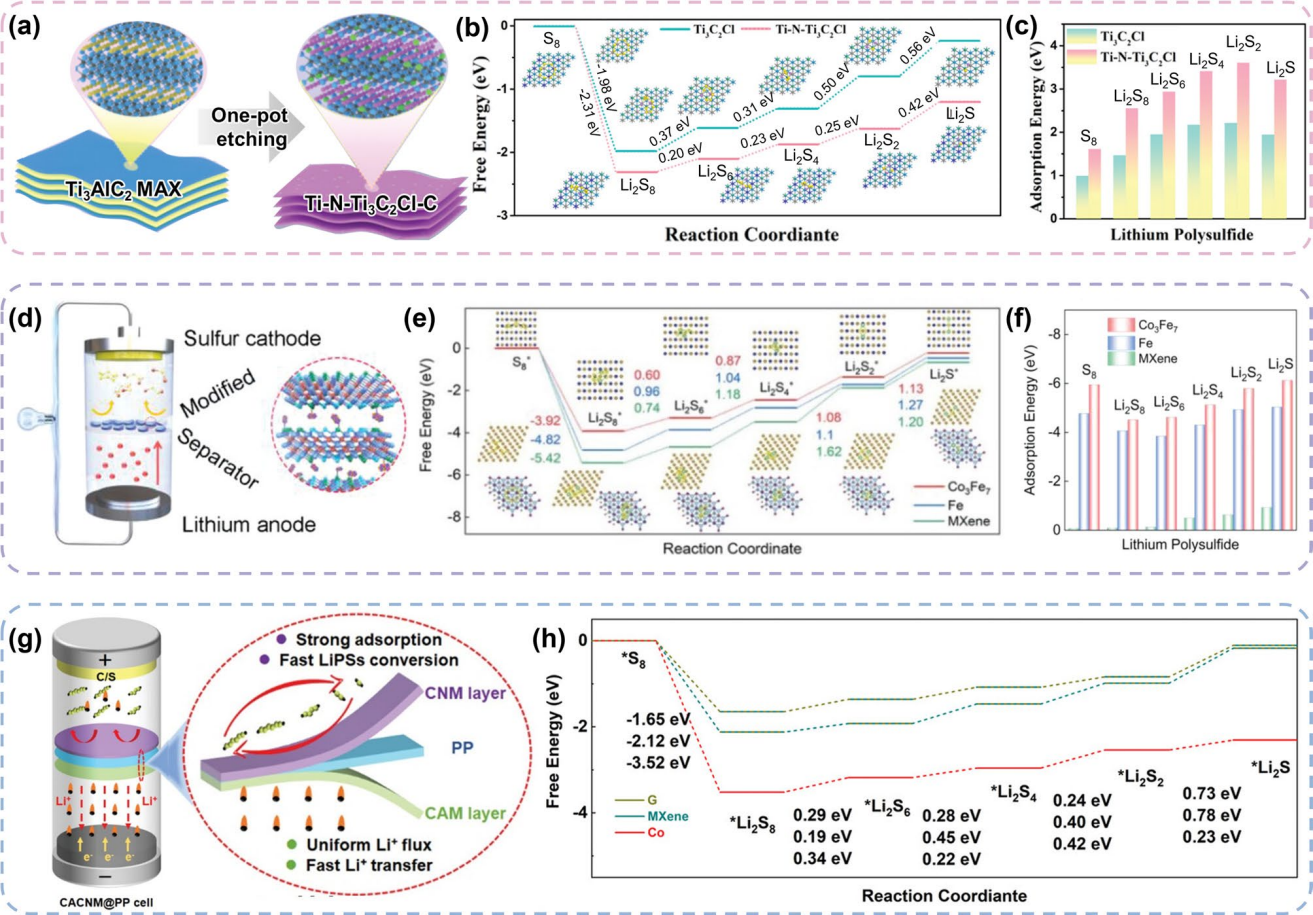
The advantages of Green MXenes for separator modification in LSBs. (**a**) Synthetic approach of the Ti-N-Ti_3_C_2_Cl-C Green MXene. Calculation results of (**b**) Gibbs free energy profiles of sulfur reduction process and (**c**) adsorption energy of LiPSs on Ti-N-Ti_3_C_2_Cl-C and Ti_3_C_2_Cl MXene. Reproduced with permission from Ref [135]. Copyright 2023, John Wiley & Sons, Inc. (**d**) Schematic illustration of the Co_3_Fe_7_-Ti_3_C_2_-modified separator and its working mechanism in suppressing the LiPS shuttle effect. (**e**) Gibbs free energy profiles of sulfur reduction process and (**f**) adsorption energy of LiPSs on Co_3_Fe_7_-Ti_3_C_2_ and conventional MXene. Reproduced with permission from Ref [136]. Copyright 2024, John Wiley & Sons, Inc. (**g**) Schematic illustration of the CACNM@PP asymmetric separator, highlighting its dual function in LiPS adsorption and Li nucleation regulation. (**h**) Comparison of Gibbs free energy calculations for the sulfur reduction process, emphasizing the catalytic advantage of the separator. Reproduced with permission from Ref [137]. Copyright 2024, John Wiley & Sons, Inc

Green MXenes have also been used as separator coating materials in LSBs to capture LiPS and promote sulfur conversion reactions. Feng et al. proposed a functional separator coated with a hierarchical porous N-doped carbon (HPNC)-encapsulated fluorine-free MXene (Ti-N-Ti_3_C_2_Cl-C) using Lewis acid molten salt method (Fig. 10a) [135]. This unique design demonstrated a strong capability to facilitate the conversion reactions of the sulfur cathode (Fig. 10b) and the adsorption of LiPSs (Fig. 10c). This was attributed to the combined chemisorption effects of the Ti-N active sites and the enhanced micro- and mesoporosity provided by the HPNC. Consequently, the nanohybrid architecture effectively immobilized and converted the LiPSs, thereby addressing the common limitations of LSBs. Electrochemical cells with the Ti-N-Ti3C2Cl-C@PP separator exhibited a higher exchange current density (12.0 mA cm^− 2^) and lower overpotential (0.104 V) than the conventional PP separator (0.199 V). Similarly, the same group designed a fluoride-free Ti_3_C_2_ MXene with a Co_3_Fe_7_ bimetallic alloy (Co_3_Fe_7_-MXene) *via* a one-pot molten salt etching strategy [136]. Sub-nanosized Co_3_Fe_7_ particles were in-situ formed and tightly anchored on MXene. As illustrated in Fig. 10d, this customized Co_3_Fe_7_-MXene was coated onto the separator and functioned as a barrier to inhibit the LiPS shuttle effect. It was reported that the bimetallic electronic structure of Co and Fe resulted in higher catalytic activity (Fig. 10e), along with a stronger LiPS anchoring capability (Fig. 10f**)**. The Co_3_Fe_7_-MXene-coated separator exhibited a capacity retention of 59.1% after 500 cycles, demonstrating improved reversibility compared with the pristine MXene-coated separator (43%) in full-cell tests.

Meng et al. introduced an MXene-bimetallic hybrid asymmetric separator (CACNM@PP), modifying both sides of separator with different green MXenes. It is composed of Co–Ni/MXene (CNM) on the cathode side and Cu–Ag/MXene (CAM) on the Li anode side (Fig. 10g) [137]. On the cathode side, CNM provided strong adsorption and rapid LiPS conversion with a synergistic effect by integrating Co, Ni, and MXene (Fig. 10h). For the anode, abundant lithiophilic sites and negatively charged surfaces were expected to lower the nucleation barrier of Li and induce a uniform Li-ion flux. Moreover, an inorganic LiCl/LiF SEI layer was shown to form, providing stable Li-electrolyte interface during charging and discharging. As a result, on the anode side, the Li metal symmetric cell exhibited a low overpotential and high cyclability, whereas on the cathode side, high sulfur utilization and effective suppression of the LiPS shuttle effect were observed.

It is evident that green MXenes potentially provide several advantages for separator modification in LSBs. Nonetheless, these approaches require meticulous attention because coating conductive materials on separator may result in internal short circuits. Additionally, this modification may impede Li-ion transport due to increased tortuosity of the separator. Furthermore, the mechanical integrity and long-term stability of these coatings should be optimized to prevent layer detachment and degradation during extended cycling. Nevertheless, the high versatility of green MXenes makes these highly potential as functional additives for separator in future LSBs.

## Outlook and perspective

**Fig. 11:**
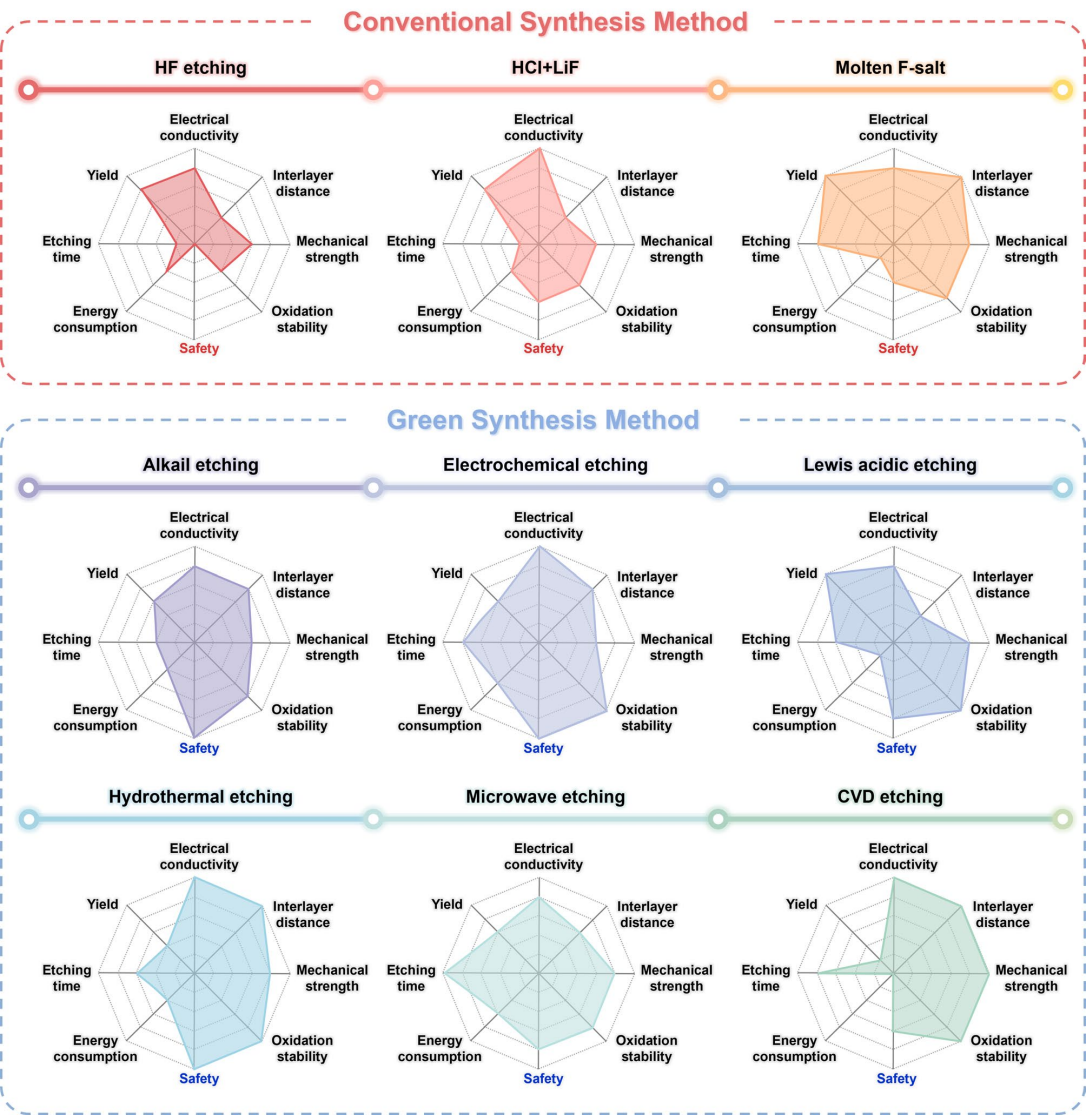
Characteristics of Conventional and green synthesis methods. The Radar plots of comprehensive comparison of conventional synthesis method: HF etching, HCl + LiF, Molten F-salt and green synthesis method: Alkail etching, electrochemical etching, Lewis acidic etching, hydrothermal etching, microwave etching, CVD etching

Figure 11 provides a comparative overview of existing MXene synthesis methods using radar plots to visualize key performance attributes. For example, safety is assessed based on the toxicity of the etchants and the operational risks. Energy consumption is evaluated in terms of reaction temperature and duration (e.g., molten salt methods operating above 750 °C for up to 24 h). Oxidation stability and interlayer distance are estimated from reported surface terminations and flake morphology. From a materials perspective, properties such as electrical conductivity, interlayer spacing, mechanical integrity, and oxidation stability play a critical role in determining electrochemical behavior. From a processing standpoint, factors including synthesis yield, etching time, energy consumption, and safety must also be carefully considered. These parameters vary substantially depending on the synthesis route and directly influence the performance and scalability of MXenes in battery applications.

In this context, green synthesis methods-particularly those that avoid the use of hydrofluoric acid (HF)-have emerged as promising alternatives to conventional fluorine-based approaches. These methods offer clear advantages in terms of safety, environmental sustainability, and compatibility with industrial practices. Moreover, green MXenes often exhibit superior intrinsic properties, such as improved stability and tunable surface chemistry, which are beneficial for enhancing battery performance. Nonetheless, green synthesis routes are not without their challenges: issues such as relatively low yield, extended processing time, and higher energy input remain significant hurdles to commercial scalability. Overcoming these barriers is crucial for the widespread adoption of MXenes in next-generation energy storage systems. The evolution of green MXene synthesis marks a pivotal advancement toward the environmentally conscious and scalable production of these highly promising 2D materials. This review has outlined the trajectory from early HF-based etching techniques to more sustainable, HF-free strategies, while connecting each synthesis method to its practical implications for lithium-ion and post-lithium-ion battery technologies.

Looking ahead, the functional role of green MXenes is expected to expand well beyond that of conventional conductive additives. Their unique physicochemical versatility opens new opportunities for interface engineering, ion transport regulation, and structural stabilization within complex battery architectures. However, to fully leverage their potential, researchers must carefully evaluate and select synthesis approaches based on specific material requirements, system constraints, and application targets. As the field progresses, maintaining a strong focus on green and scalable synthesis will be essential-not only for performance optimization but also for aligning battery innovation with global sustainability goals.

## Data Availability

The datasets used and/or analyzed in the current study are available from the corresponding author upon reasonable request.

## References

[1] J. Janek, W.G. Zeier, A solid future for battery development. Nat. Energy. **1**, 16141 (2016). 10.1038/nenergy.2016.141

[2] T. Placke, R. Kloepsch, S. Dühnen, M. Winter, Lithium ion, lithium metal, and alternative rechargeable battery technologies: the odyssey for high energy density. J. Solid State Electrochem. **21**, 1939 (2017). 10.1007/s10008-017-3610-7

[3] J. Li, Z. Du, R.E. Ruther, S.J. An, L.A. David, K. Hays, M. Wood, N.D. Phillip, Y. Sheng, C. Mao, S. Kalnaus, C. Daniel, D.L. Wood, Toward Low-Cost, High-Energy density, and High-Power density Lithium-Ion batteries. JOM. **69**, 1484 (2017). 10.1007/s11837-017-2404-9

[4] J. Liu, Z. Bao, Y. Cui, E.J. Dufek, J.B. Goodenough, P. Khalifah, Q. Li, B.Y. Liaw, P. Liu, A. Manthiram, Y.S. Meng, V.R. Subramanian, M.F. Toney, V.V. Viswanathan, M.S. Whittingham, J. Xiao, W. Xu, J. Yang, X.-Q. Yang, J.-G. Zhang, Pathways for practical high-energy long-cycling lithium metal batteries. Nat. Energy. **4**, 180 (2019). 10.1038/s41560-019-0338-x

[5] J. Kim, M. Kim, J. Lee, J. An, S. Yang, H.C. Ahn, D.-J. Yoo, J.W. Choi, Insights from Li and Zn systems for advancing Mg and Ca metal batteries. Chem. Soc. Rev. **53**, 8878 (2024). 10.1039/D4CS00557K 39106108 10.1039/d4cs00557k

[6] H.R. Oliveira Filho, H. Zanin, R.S. Monteiro, M.H.P. Barbosa, R.F. Teófilo, High–nickel cathodes for lithium-ion batteries: from synthesis to electricity. J. Energy Storage. **82**, 110536 (2024). 10.1016/j.est.2024.110536

[7] P. Vanaphuti, Z. Cui, A. Manthiram, Demarcating the impact of electrolytes on High-Nickel cathodes and Lithium-Metal anode. Adv. Funct. Mater. **34**, 2308619 (2024). 10.1002/adfm.202308619. (acccessed 2025/01/15)

[8] A. Manthiram, A reflection on lithium-ion battery cathode chemistry. Nat. Commun. **11**, 1550 (2020). 10.1038/s41467-020-15355-0 32214093 10.1038/s41467-020-15355-0 PMC7096394

[9] K. Kang, Y.S. Meng, J. Bréger, C.P. Grey, G. Ceder, Electrodes with high power and high capacity for rechargeable Lithium batteries. Science. **311**, 977 (2006). 10.1126/science.1122152. (acccessed 2025/06/04) 16484487 10.1126/science.1122152

[10] H. Huo, M. Jiang, Y. Bai, S. Ahmed, K. Volz, H. Hartmann, A. Henss, C.V. Singh, D. Raabe, J. Janek, Chemo-mechanical failure mechanisms of the silicon anode in solid-state batteries. Nat. Mater. **23**, 543 (2024). 10.1038/s41563-023-01792-x 38278984 10.1038/s41563-023-01792-x PMC10990934

[11] P. Li, H. Kim, S.-T. Myung, Y.-K. Sun, Diverting exploration of silicon anode into practical way: A review focused on silicon-Graphite composite for Lithium ion batteries. Energy Storage Mater. **35**, 550 (2021). 10.1016/j.ensm.2020.11.028

[12] L. Sun, Y. Liu, R. Shao, J. Wu, R. Jiang, Z. Jin, Recent progress and future perspective on practical silicon anode-based lithium ion batteries. Energy Storage Mater. **46**, 482 (2022). 10.1016/j.ensm.2022.01.042

[13] H. Jo, J.-W. Lee, E. Kwon, S. Yu, B.G. Kim, S. Park, J. Moon, M.J. Ko, Lim, electrochemically tailored host design with gradient seeds for Dendrite-Free Li metal batteries. ACS Nano. **18**, 35718 (2024). 10.1021/acsnano.4c15556 39688156 10.1021/acsnano.4c15556

[14] S. Won, A. Jung, K.Y. Lim, J. Cho, J.G. Son, H.-D. Lim, B. Yeom, Layer-by-Layer assembly of graphene oxide and silver nanowire thin films with interdigitated nanostructure in dendrite suppressions of Li-Metal batteries. Small. **21**, 2412784 (2025). 10.1002/smll.202412784. (acccessed 2025/03/16) 39901500 10.1002/smll.202412784 PMC11922020

[15] S. Kim, S. Lee, H.S. Ryu, H. Jo, J. Yun, S. Mun, S. Park, K. Kim, H.-D. Lim, Fluorine-aligned functional MXene enabling unusual bead-like Li growth for anode-less Li-metal batteries. Chem. Eng. J. **513**, 162294 (2025). 10.1016/j.cej.2025.162294

[16] S. Mun, S. Kim, J. Yun, H.S. Ryu, S. Park, H. Jo, H.-D. Lim, Tailored Fluorine-Rich MXene with interlayer architecture for enhanced stability in Anode-Free Lithium metal batteries. ACS Appl. Energy Mater. **8**, 6474 (2025)

[17] D. Lin, Y. Liu, Y. Cui, Reviving the lithium metal anode for high-energy batteries. Nat. Nanotechnol. **12**, 194 (2017). 10.1038/nnano.2017.16 28265117 10.1038/nnano.2017.16

[18] G. Zhou, H. Chen, Y. Cui, Formulating energy density for designing practical lithium–sulfur batteries. Nat. Energy. **7**, 312 (2022). 10.1038/s41560-022-01001-0

[19] G.G. Eshetu, H. Zhang, X. Judez, H. Adenusi, M. Armand, S. Passerini, E. Figgemeier, Production of high-energy Li-ion batteries comprising silicon-containing anodes and insertion-type cathodes. Nat. Commun. **12**, 5459 (2021). 10.1038/s41467-021-25334-8 34526508 10.1038/s41467-021-25334-8 PMC8443554

[20] D. Eum, B. Kim, S.J. Kim, H. Park, J. Wu, S.-P. Cho, G. Yoon, M.H. Lee, S.-K. Jung, W. Yang, W.M. Seong, K. Ku, O. Tamwattana, S.K. Park, I. Hwang, K. Kang, Voltage decay and redox asymmetry mitigation by reversible cation migration in lithium-rich layered oxide electrodes. Nat. Mater. **19**, 419 (2020). 10.1038/s41563-019-0572-4 31959949 10.1038/s41563-019-0572-4

[21] J.W. Choi, D. Aurbach, Promise and reality of post-lithium-ion batteries with high energy densities. Nat. Rev. Mater. **1**, 16013 (2016). 10.1038/natrevmats.2016.13

[22] X.-B. Cheng, R. Zhang, C.-Z. Zhao, Q. Zhang, Toward safe Lithium metal anode in rechargeable batteries: A review. Chem. Rev. **117**, 10403 (2017). 10.1021/acs.chemrev.7b00115 28753298 10.1021/acs.chemrev.7b00115

[23] H. Kwon, J.-H. Lee, Y. Roh, J. Baek, D.J. Shin, J.K. Yoon, H.J. Ha, J.Y. Kim, H.-T. Kim, An electron-deficient carbon current collector for anode-free Li-metal batteries. Nat. Commun. **12**, 5537 (2021). 10.1038/s41467-021-25848-1 34545077 10.1038/s41467-021-25848-1 PMC8452779

[24] X. Shen, Z. Tian, R. Fan, L. Shao, D. Zhang, G. Cao, L. Kou, Y. Bai, Research progress on silicon/carbon composite anode materials for lithium-ion battery. J. Energy Chem. **27**, 1067 (2018). 10.1016/j.jechem.2017.12.012

[25] Z. Li, Y. Huang, L. Yuan, Z. Hao, Y. Huang, Status and prospects in sulfur–carbon composites as cathode materials for rechargeable lithium–sulfur batteries. Carbon. **92**, 41 (2015). 10.1016/j.carbon.2015.03.008

[26] R. Xu, X.-Q. Zhang, X.-B. Cheng, H.-J. Peng, C.-Z. Zhao, C. Yan, J.-Q. Huang, Artificial Soft–Rigid protective layer for Dendrite-Free Lithium metal anode. Adv. Funct. Mater. **28**, 1705838 (2018). 10.1002/adfm.201705838. (acccessed 2025/06/04)

[27] J. Hwang, S. Lee, S. Kim, K. Do, S. Kim, H. Jo, H.-D. Lim, H. Ahn, Uniform and multifunctional PEI-POSS/Carbon encapsulation for High-Rate performance and surface stabilization of Nickel-Rich layered cathodes in Lithium-Ion batteries. Adv. Funct. Mater. **33**, 2304614 (2023). 10.1002/adfm.202304614. (acccessed 2025/06/04)

[28] K. Kim, H. Ma, S. Park, N.-S. Choi, Electrolyte-Additive-Driven interfacial engineering for High-Capacity electrodes in Lithium-Ion batteries: promise and challenges. ACS Energy Lett. **5**, 1537 (2020). 10.1021/acsenergylett.0c00468

[29] S. Moon, H. Park, G. Yoon, M.H. Lee, K.-Y. Park, K. Kang, Simple and effective Gas-Phase doping for Lithium metal protection in Lithium metal batteries. Chem. Mater. **29**, 9182 (2017). 10.1021/acs.chemmater.7b03027

[30] H. Yuan, X. Ding, T. Liu, J. Nai, Y. Wang, Y. Liu, C. Liu, X. Tao, A review of concepts and contributions in lithium metal anode development. Mater. Today. **53**, 173 (2022). 10.1016/j.mattod.2022.01.015

[31] A.D. Pathak, E. Cha, W. Choi, Towards the commercialization of Li-S battery: from lab to industry. Energy Storage Mater. **72**, 103711 (2024). 10.1016/j.ensm.2024.103711

[32] M. Endo, C. Kim, K. Nishimura, T. Fujino, K. Miyashita, Recent development of carbon materials for Li ion batteries. Carbon. **38**, 183 (2000). 10.1016/S0008-6223(99)00141-4

[33] S. Flandrois, B. Simon, Carbon materials for lithium-ion rechargeable batteries. Carbon. **37**, 165 (1999). 10.1016/S0008-6223(98)00290-5

[34] C. de las Casas, W. Li, A review of application of carbon nanotubes for lithium ion battery anode material. J. Power Sources. **208**, 74 (2012). 10.1016/j.jpowsour.2012.02.013

[35] X.-Q. Zhang, X.-B. Cheng, X. Chen, C. Yan, Q. Zhang, Fluoroethylene carbonate additives to render uniform Li deposits in Lithium metal batteries. Adv. Funct. Mater. **27**, 1605989 (2017). 10.1002/adfm.201605989. (acccessed 2025/06/04)

[36] S. Stuckenberg, M.M. Bela, C.-T. Lechtenfeld, M. Mense, V. Küpers, T.T.K. Ingber, M. Winter, M.C. Stan, Influence of LiNO3 on the Lithium metal deposition behavior in Carbonate-Based liquid electrolytes and on the electrochemical performance in Zero-Excess Lithium metal batteries. Small. **20**, 2305203 (2024). 10.1002/smll.202305203. (acccessed 2025/06/04) 10.1002/smll.202305203 37797185

[37] X. Li, Z. Huang, C.E. Shuck, G. Liang, Y. Gogotsi, C. Zhi, MXene chemistry, electrochemistry and energy storage applications. Nat. Rev. Chem. **6**, 389 (2022). 10.1038/s41570-022-00384-8 37117426 10.1038/s41570-022-00384-8

[38] S. Mun, S. Kim, J. Yun, H.S. Ryu, S. Park, H. Jo, H.-D. Lim, Tailored Fluorine-Rich MXene with interlayer architecture for enhanced stability in Anode-Free Lithium metal batteries. ACS Appl. Energ. Mater. **8**, 6474 (2025). 10.1021/acsaem.5c00292

[39] M. Naguib, M. Kurtoglu, V. Presser, J. Lu, J. Niu, M. Heon, L. Hultman, Y. Gogotsi, M.W. Barsoum, Two-Dimensional nanocrystals produced by exfoliation of Ti3AlC2. Adv. Mater. **23**, 4248 (2011). 10.1002/adma.201102306 21861270 10.1002/adma.201102306

[40] A. Szuplewska, D. Kulpińska, A. Dybko, A.M. Jastrzębska, T. Wojciechowski, A. Rozmysłowska, M. Chudy, I. Grabowska-Jadach, W. Ziemkowska, Z. Brzózka, A. Olszyna, 2D Ti2C (MXene) as a novel highly efficient and selective agent for photothermal therapy. Mater. Sci. Eng. C **98**, 874 (2019). 10.1016/j.msec.2019.01.021 10.1016/j.msec.2019.01.021 30813093

[41] F. Liu, J. Zhou, S. Wang, B. Wang, C. Shen, L. Wang, Q. Hu, Q. Huang, A. Zhou, Preparation of High-Purity V2C MXene and electrochemical properties as Li-Ion batteries. J. Electrochem. Soc. **164**, A709 (2017). 10.1149/2.0641704jes

[42] R. Meshkian, Q. Tao, M. Dahlqvist, J. Lu, L. Hultman, J. Rosen, Theoretical stability and materials synthesis of a chemically ordered MAX phase, Mo2ScAlC2, and its two-dimensional derivate Mo2ScC2 MXene. Acta Mater. **125**, 476 (2017). 10.1016/j.actamat.2016.12.008

[43] A. Lakmal, P.B. Thombre, C.E. Shuck, Solid-Solution mxenes: synthesis, properties, and applications. Acc. Chem. Res. **57**, 3007 (2024). 10.1021/acs.accounts.4c00387 39357063 10.1021/acs.accounts.4c00387

[44] Y. Guan, L. Jiang, R. Zhao, X. Li, H. Zhu, Q. Zhang, Z. Dong, N. Yang, Y. Cong, Ti2TaC2: A novel out-of-plane ordered MXene towards flexible and cytocompatible supercapacitors. Chem. Eng. J. **496**, 154082 (2024). 10.1016/j.cej.2024.154082

[45] J. Yang, R. Liu, N. Jia, K. Wu, X. Fu, Q. Wang, W. Cui, Novel W-based in-plane chemically ordered (W2/3R1/3)2AlC (R = Gd, tb, dy, ho, er, Tm and Lu) MAX phases and their 2D W1.33 C MXene derivatives. Carbon. **183**, 76 (2021). 10.1016/j.carbon.2021.07.010

[46] S.K. Nemani, B. Zhang, B.C. Wyatt, Z.D. Hood, S. Manna, R. Khaledialidusti, W. Hong, M.G. Sternberg, S.K.R.S. Sankaranarayanan, B. Anasori, High-Entropy 2D carbide mxenes: TiVNbMoC3 and TiVCrMoC3. ACS Nano. **15**, 12815 (2021). 10.1021/acsnano.1c02775 34128649 10.1021/acsnano.1c02775

[47] V. Natu, M.W. Barsoum, MXene surface terminations: A perspective. J. Phys. Chem. C **127**, 20197 (2023). 10.1021/acs.jpcc.3c04324

[48] S. Wang, Y. Liu, Y. Liu, W. Hu, Effect of HF etching on titanium carbide (Ti3C2Tx) microstructure and its capacitive properties. Chem. Eng. J. **452**, 139512 (2023). 10.1016/j.cej.2022.139512

[49] M. Naguib, O. Mashtalir, J. Carle, V. Presser, J. Lu, L. Hultman, Y. Gogotsi, M.W. Barsoum, Two-dimensional transition metal carbides. ACS Nano. **6**, 1322 (2012) 22279971 10.1021/nn204153h

[50] M. Anayee, C.E. Shuck, M. Shekhirev, A. Goad, R. Wang, Y. Gogotsi, Kinetics of Ti3AlC2 etching for Ti3C2T x MXene synthesis. Chem. Mater. **34**, 9589 (2022)

[51] M. Alhabeb, K. Maleski, B. Anasori, P. Lelyukh, L. Clark, S. Sin, Y. Gogotsi, Guidelines for synthesis and processing of Two-Dimensional titanium carbide (Ti3C2Tx MXene). Chem. Mater. **29**, 7633 (2017). 10.1021/acs.chemmater.7b02847

[52] M. Saraf, B. Chacon, S. Ippolito, R.W. Lord, M. Anayee, R. Wang, A. Inman, C.E. Shuck, Y. Gogotsi, Enhancing charge storage of Mo2Ti2C3 MXene by partial oxidation. Adv. Funct. Mater. **34**, 2306815 (2024). 10.1002/adfm.202306815. (acccessed 2025/06/04)

[53] M. Dahlqvist, J. Zhou, I. Persson, B. Ahmed, J. Lu, J. Halim, Q. Tao, J. Palisaitis, J. Thörnberg, P. Helmer, L. Hultman, P.O.Å. Persson, J. Rosen, Out-Of-Plane ordered laminate borides and their 2D Ti-Based derivative from chemical exfoliation. Adv. Mater. **33**, 2008361 (2021). 10.1002/adma.202008361. (acccessed 2025/06/04) 34350624 10.1002/adma.202008361 PMC11468983

[54] M.R. Lukatskaya, O. Mashtalir, C.E. Ren, Y. Dall’Agnese, P. Rozier, P.L. Taberna, M. Naguib, P. Simon, M.W. Barsoum, Y. Gogotsi, Cation intercalation and high volumetric capacitance of Two-Dimensional titanium carbide. Science. **341**, 1502 (2013). 10.1126/science.1241488. (acccessed 2025/06/04) 24072919 10.1126/science.1241488

[55] P. Lakhe, E.M. Prehn, T. Habib, J.L. Lutkenhaus, M. Radovic, M.S. Mannan, M.J. Green, Process safety analysis for Ti3C2Tx MXene synthesis and processing. Ind. Eng. Chem. Res. **58**, 1570 (2019). 10.1021/acs.iecr.8b05416

[56] X. Sang, Y. Xie, M.-W. Lin, M. Alhabeb, K.L. Van Aken, Y. Gogotsi, P.R.C. Kent, K. Xiao, R.R. Unocic, Atomic defects in monolayer titanium carbide (Ti3C2Tx) MXene. ACS Nano. **10**, 9193 (2016). 10.1021/acsnano.6b05240 27598326 10.1021/acsnano.6b05240

[57] S. Yang, P. Zhang, F. Wang, A.G. Ricciardulli, M.R. Lohe, P.W.M. Blom, X. Feng, Fluoride-Free synthesis of Two-Dimensional titanium carbide (MXene) using A binary aqueous system. Angew Chem. Int. Edit. **57**, 15491 (2018). 10.1002/anie.201809662. (acccessed 2025/06/04) 10.1002/anie.201809662 30289581

[58] M. Ghidiu, M.R. Lukatskaya, M.-Q. Zhao, Y. Gogotsi, M.W. Barsoum, Conductive two-dimensional titanium carbide ‘clay’ with high volumetric capacitance. Nature. **516**, 78 (2014). 10.1038/nature13970 25470044 10.1038/nature13970

[59] M. Ghidiu, M.R. Lukatskaya, M.-Q. Zhao, Y. Gogotsi, M.W. Barsoum, Conductive two-dimensional titanium carbide ‘clay’with high volumetric capacitance. Nature. **516**, 78 (2014) 25470044 10.1038/nature13970

[60] T. Zhang, L. Pan, H. Tang, F. Du, Y. Guo, T. Qiu, J. Yang, Synthesis of two-dimensional Ti3C2Tx MXene using HCl + LiF etchant: enhanced exfoliation and delamination. J. Alloy Compd. **695**, 818 (2017). 10.1016/j.jallcom.2016.10.127

[61] A. Lipatov, M. Alhabeb, M.R. Lukatskaya, A. Boson, Y. Gogotsi, A. Sinitskii, Effect of synthesis on quality, electronic properties and environmental stability of individual monolayer Ti3C2 MXene flakes. Adv. Electron. Mater. **2**, 1600255 (2016)

[62] J. Zhang, N. Kong, S. Uzun, A. Levitt, S. Seyedin, P.A. Lynch, S. Qin, M. Han, W. Yang, J. Liu, X. Wang, Y. Gogotsi, J.M. Razal, Scalable manufacturing of Free-Standing, strong Ti3C2Tx MXene films with outstanding conductivity. Adv. Mater. **32**, 2001093 (2020). 10.1002/adma.202001093. (acccessed 2025/06/04) 10.1002/adma.202001093 32309891

[63] J. Halim, S. Kota, M.R. Lukatskaya, M. Naguib, M.-Q. Zhao, E.J. Moon, J. Pitock, J. Nanda, S.J. May, Y. Gogotsi, Barsoum, synthesis and characterization of 2D molybdenum carbide (MXene). Adv. Funct. Mater. **26**, 3118 (2016). 10.1002/adfm.201505328. (acccessed 2025/06/04)

[64] O. Mashtalir, M.R. Lukatskaya, M.-Q. Zhao, M.W. Barsoum, Y. Gogotsi, Amine-Assisted delamination of Nb2C MXene for Li-Ion energy storage devices. Adv. Mater. **27**, 3501 (2015). 10.1002/adma.201500604. (acccessed 2025/06/04) 25930685 10.1002/adma.201500604

[65] J. Halim, S. Kota, M.R. Lukatskaya, M. Naguib, M.Q. Zhao, E.J. Moon, J. Pitock, J. Nanda, S.J. May, Y. Gogotsi, Synthesis and characterization of 2D molybdenum carbide (MXene). Adv. Funct. Mater. **26**, 3118 (2016)

[66] P. Urbankowski, B. Anasori, T. Makaryan, D. Er, S. Kota, P.L. Walsh, M. Zhao, V.B. Shenoy, M.W. Barsoum, Y. Gogotsi, Synthesis of two-dimensional titanium nitride Ti 4 N 3 (MXene). Nanoscale. **8**, 11385 (2016) 27211286 10.1039/c6nr02253g

[67] F. Liu, A. Zhou, J. Chen, J. Jia, W. Zhou, L. Wang, Q. Hu, Preparation of Ti3C2 and Ti2C MXenes by fluoride salts etching and methane adsorptive properties. Appl. Surf. Sci. **416**, 781 (2017). 10.1016/j.apsusc.2017.04.239

[68] P. Urbankowski, B. Anasori, T. Makaryan, D. Er, S. Kota, P.L. Walsh, M. Zhao, V.B. Shenoy, M.W. Barsoum, Y. Gogotsi, Synthesis of two-dimensional titanium nitride Ti4N3 (MXene). Nanoscale. **8**, 11385 (2016). 10.1039/C6NR02253G 27211286 10.1039/c6nr02253g

[69] A. Thakur, N. Chandran, B.S.K. Davidson, A. Bedford, H. Fang, Y. Im, V. Kanduri, B.C. Wyatt, S.K. Nemani, V. Poliukhova, R. Kumar, Z. Fakhraai, B. Anasori, Step-by-Step guide for synthesis and delamination of Ti3C2Tx MXene. Small Methods. **7**, 2300030 (2023). 10.1002/smtd.202300030. (acccessed 2025/06/04) 10.1002/smtd.202300030 37150839

[70] O. Mashtalir, M. Naguib, V.N. Mochalin, Y. Dall’Agnese, M. Heon, M.W. Barsoum, Y. Gogotsi, Intercalation and delamination of layered carbides and carbonitrides. Nat. Commun. **4**, 1716 (2013) 23591883 10.1038/ncomms2664

[71] Y.-J. Kim, S.J. Kim, D. Seo, Y. Chae, M. Anayee, Y. Lee, Y. Gogotsi, C.W. Ahn, H.-T. Jung, Etching mechanism of monoatomic aluminum layers during MXene synthesis. Chem. Mater. **33**, 6346 (2021). 10.1021/acs.chemmater.1c01263

[72] R.A. Soomro, P. Zhang, B. Fan, Y. Wei, B. Xu, Progression in the oxidation stability of MXenes. Nano-Micro Lett. **15**, 108 (2023). 10.1007/s40820-023-01069-7 10.1007/s40820-023-01069-7 PMC10113412 37071337

[73] V. Natu, R. Pai, M. Sokol, M. Carey, V. Kalra, Barsoum, 2D Ti3C2Tz MXene synthesized by Water-free etching of Ti3AlC2 in Polar organic solvents. Chem. **6**, 616 (2020). 10.1016/j.chempr.2020.01.019

[74] K. Maleski, V.N. Mochalin, Y. Gogotsi, Dispersions of two-dimensional titanium carbide MXene in organic solvents. Chem. Mater. **29**, 1632 (2017)

[75] J. Halim, M.R. Lukatskaya, K.M. Cook, J. Lu, C.R. Smith, L.-Å. Näslund, S.J. May, L. Hultman, Y. Gogotsi, P. Eklund, Transparent conductive two-dimensional titanium carbide epitaxial thin films. Chem. Mater. **26**, 2374 (2014) 24741204 10.1021/cm500641a PMC3982936

[76] F. Wang, F. Tian, X. Xia, Z. Pang, S. Wang, X. Yu, G. Li, Y. Zhao, Q. Xu, S. Hu, L. Ji, X. Zou, X. Lu, One-step synthesis of organic terminal 2D Ti3C2Tx MXene nanosheets by etching of Ti3AlC2 in an organic Lewis acid solvent. Angew Chem. Int. Edit. **63**, e202405315 (2024). 10.1002/anie.202405315. (acccessed 2025/06/05) 10.1002/anie.202405315 38588049

[77] W. Liu, L. Li, C. Hu, D. Chen, G. Shen, Intercalation of small organic molecules into Ti3C2Tx MXene cathodes for flexible High-Volume-Capacitance Zn-Ion microsupercapacitor. Adv. Mater. Technol. **7**, 2200158 (2022). 10.1002/admt.202200158. (acccessed 2025/06/05)

[78] X. Qiu, L. Dai, H. Li, K. Qu, R. Li, Pillaring behavior of organic molecules on mxene: insights from molecular dynamics simulations. Langmuir. **39**, 14912 (2023). 10.1021/acs.langmuir.3c01682 37812693 10.1021/acs.langmuir.3c01682

[79] Z. Wang, H. Jiang, Y. Zhang, Y. An, C. Wei, L. Tan, S. Xiong, Y. Qian, J. Feng, Application of 2D MXene in organic electrode materials for rechargeable batteries: recent progress and perspectives. Adv. Funct. Mater. **33**, 2210184 (2023). 10.1002/adfm.202210184. (acccessed 2025/06/05)

[80] L. Cseri, M. Razali, P. Pogany, G. Szekely, Chapter 3.15 - Organic solvents in sustainable synthesis and engineering, in *Green Chemistry*, ed. by B. Török, T. Dransfield (Elsevier, 2018), p. 513

[81] L. Liu, M. Orbay, S. Luo, S. Duluard, H. Shao, J. Harmel, P. Rozier, P.-L. Taberna, P. Simon, Exfoliation and delamination of Ti3C2Tx MXene prepared via molten salt etching route. ACS Nano. **16**, 111 (2022). 10.1021/acsnano.1c08498 34787390 10.1021/acsnano.1c08498

[82] T. Li, L. Yao, Q. Liu, J. Gu, R. Luo, J. Li, X. Yan, W. Wang, P. Liu, B. Chen, Fluorine-free synthesis of high‐purity Ti3C2Tx (T = OH, O) via alkali treatment. Angew Chem. Int. Edit. **57**, 6115 (2018) 10.1002/anie.201800887 29633442

[83] G. Li, L. Tan, Y. zhang, B. Wu, L. Li, Highly efficiently delaminated Single-Layered MXene nanosheets with large lateral size. Langmuir. **33**, 9000 (2017). 10.1021/acs.langmuir.7b01339 28805394 10.1021/acs.langmuir.7b01339

[84] K.R.G. Lim, M. Shekhirev, B.C. Wyatt, B. Anasori, Y. Gogotsi, Z.W. Seh, Fundamentals of MXene synthesis. Nat. Synth. **1**, 601 (2022). 10.1038/s44160-022-00104-6

[85] V. Natu, R. Pai, O. Wilson, E. Gadasu, H. Badr, A. Karmakar, A.J.D. Magenau, V. Kalra, M.W. Barsoum, Effect of base/nucleophile treatment on interlayer ion intercalation, surface terminations, and osmotic swelling of Ti3C2Tz MXene. Multilayers Chem. Mater. **34**, 678 (2022). 10.1021/acs.chemmater.1c03390

[86] K. Montazeri, H. Badr, K. Ngo, K. Sudhakar, T. Elmelegy, J. Uzarski, V. Natu, M.W. Barsoum, Delamination of MXene flakes using simple inorganic bases. J. Phys. Chem. C **127**, 10391 (2023). 10.1021/acs.jpcc.3c02318

[87] W. Sun, S.A. Shah, Y. Chen, Z. Tan, H. Gao, T. Habib, M. Radovic, M.J. Green, Electrochemical etching of Ti2AlC to Ti2CTx (MXene) in low-concentration hydrochloric acid solution. J. Mater. Chem. A **5**, 21663 (2017). 10.1039/C7TA05574A

[88] L. Liu, H. Zschiesche, M. Antonietti, M. Gibilaro, P. Chamelot, L. Massot, P. Rozier, P.-L. Taberna, P. Simon, In situ synthesis of MXene with tunable morphology by electrochemical etching of MAX phase prepared in molten salt. Adv. Energy Mater. **13**, 2203805 (2023). 10.1002/aenm.202203805. (acccessed 2025/06/04)

[89] W. Sun, S. Shah, Y. Chen, Z. Tan, H. Gao, T. Habib, M. Radovic, M. Green, Electrochemical etching of Ti 2 AlC to Ti 2 CT x (MXene) in low-concentration hydrochloric acid solution. J. Mater. Chem. A **5**, 21663 (2017)

[90] T. Yin, Y. Li, R. Wang, O.A. Al-Hartomy, A. Al-Ghamdi, S. Wageh, X. Luo, X. Tang, H. Zhang, Synthesis of Ti3C2Fx MXene with controllable fluorination by electrochemical etching for lithium-ion batteries applications. Ceram. Int. **47**, 28642 (2021). 10.1016/j.ceramint.2021.07.023

[91] S.-Y. Pang, Y.-T. Wong, S. Yuan, Y. Liu, M.-K. Tsang, Z. Yang, H. Huang, W.-T. Wong, J. Hao, Universal strategy for HF-Free facile and rapid synthesis of Two-dimensional MXenes as multifunctional energy materials. J. Am. Chem. Soc. **141**, 9610 (2019). 10.1021/jacs.9b02578 31117483 10.1021/jacs.9b02578

[92] M.P. Bilibana, Electrochemical properties of MXenes and applications. Adv. Sens. Energy Mater. **2**, 100080 (2023). 10.1016/j.asems.2023.100080

[93] M. Li, J. Lu, K. Luo, Y. Li, K. Chang, K. Chen, J. Zhou, J. Rosen, L. Hultman, P. Eklund, P.O.Å. Persson, S. Du, Z. Chai, Z. Huang, Q. Huang, Element replacement approach by reaction with Lewis acidic molten salts to synthesize nanolaminated MAX phases and MXenes. J. Am. Chem. Soc. **141**, 4730 (2019). 10.1021/jacs.9b00574 30821963 10.1021/jacs.9b00574

[94] M. Li, J. Lu, K. Luo, Y. Li, K. Chang, K. Chen, J. Zhou, J. Rosen, L. Hultman, P. Eklund, Element replacement approach by reaction with Lewis acidic molten salts to synthesize nanolaminated MAX phases and MXenes. J. Am. Chem. Soc. **141**, 4730 (2019) 30821963 10.1021/jacs.9b00574

[95] H. Wei, L. Chen, H. Ding, Y. Li, Z. Chai, Q. Huang, Dual-Phase structure through selective etching of the double A-Element MAX phase in Lewis acidic molten salts. J. Phys. Chem. Lett. **15**, 4486 (2024). 10.1021/acs.jpclett.4c00785 38634523 10.1021/acs.jpclett.4c00785

[96] X. Liu, Y. Li, H. Ding, L. Chen, S. Du, Z. Chai, Q. Huang, Topotactic transition of Ti4AlN3 MAX phase in Lewis acid molten salt. J. Materiomics. **9**, 1032 (2023). 10.1016/j.jmat.2023.03.012

[97] Y. Li, H. Shao, Z. Lin, J. Lu, L. Liu, B. Duployer, P.O.Å. Persson, P. Eklund, L. Hultman, M. Li, K. Chen, X.-H. Zha, S. Du, P. Rozier, Z. Chai, E. Raymundo-Piñero, P.-L. Taberna, P. Simon, Q. Huang, A general Lewis acidic etching route for Preparing MXenes with enhanced electrochemical performance in non-aqueous electrolyte. Nat. Mater. **19**, 894 (2020). 10.1038/s41563-020-0657-0 32284597 10.1038/s41563-020-0657-0

[98] E. Defoy, M. Baron, A. Séné, A. Ghoridi, D. Thiaudière, S. Célérier, P. Chartier, F. Brette, V. Mauchamp, D. Portehault, Galvanic replacement and etching of MAX-related phases in molten salts toward mxenes: an in situ study. Chem. Mater. **35**, 8112 (2023)

[99] K. Arole, J.W. Blivin, A.M. Bruce, S. Athavale, I.J. Echols, H. Cao, Z. Tan, M. Radovic, J.L. Lutkenhaus, M.J. Green, Exfoliation, delamination, and oxidation stability of molten salt etched Nb2CTz MXene nanosheets. Chem. Commun. **58**, 10202 (2022). 10.1039/D2CC02237K 10.1039/d2cc02237k 36000425

[100] H. Dong, P. Xiao, N. Jin, B. Wang, Y. Liu, Z. Lin, Molten salt derived Nb2CTx MXene anode for Li-ion batteries. ChemElectroChem. **8**, 957 (2021). 10.1002/celc.202100142. (acccessed 2025/06/05)

[101] P. Huang, H. Ying, S. Zhang, Z. Zhang, W.-Q. Han, Molten salts etching route driven universal construction of mxene/transition metal sulfides heterostructures with interfacial electronic coupling for superior sodium storage. Adv. Energy Mater. **12**, 2202052 (2022). 10.1002/aenm.202202052. (acccessed 2025/06/05)

[102] D.D. Kruger, H. García, A. Primo, Molten salt derived mxenes: synthesis and applications. Adv. Sci. **11**, 2307106 (2024). 10.1002/advs.202307106. (acccessed 2025/06/05) 10.1002/advs.202307106 PMC11425216 39021320

[103] J. Mei, G.A. Ayoko, C. Hu, J.M. Bell, Z. Sun, Two-dimensional fluorine-free mesoporous Mo2C MXene via UV-induced selective etching of Mo2Ga2C for energy storage. Sustain. Mater. Technol. **25**, e00156 (2020)

[104] T. Thomas, S. Pushpan, J.A. Aguilar Martínez, A. Torres Castro, N. Pineda Aguilar, A. Álvarez-Méndez, K.C. Sanal, UV-assisted safe etching route for the synthesis of Mo2CTx MXene from Mo–In–C non-MAX phase. Ceram. Int. **47**, 35384 (2021). 10.1016/j.ceramint.2021.08.342

[105] Y. Guo, S. Jin, L. Wang, P. He, Q. Hu, L.-Z. Fan, A. Zhou, Synthesis of two-dimensional carbide Mo2CTx MXene by hydrothermal etching with fluorides and its thermal stability. Ceram. Int. **46**, 19550 (2020). 10.1016/j.ceramint.2020.05.008

[106] C. Peng, P. Wei, X. Chen, Y. Zhang, F. Zhu, Y. Cao, H. Wang, H. Yu, F. Peng, A hydrothermal etching route to synthesis of 2D MXene (Ti3C2, Nb2C): enhanced exfoliation and improved adsorption performance. Ceram. Int. **44**, 18886 (2018). 10.1016/j.ceramint.2018.07.124

[107] C. Wang, H. Shou, S. Chen, S. Wei, Y. Lin, P. Zhang, Z. Liu, K. Zhu, X. Guo, X. Wu, HCl-based hydrothermal etching strategy toward fluoride‐free MXenes. Adv. Mater. **33**, 2101015 (2021) 10.1002/adma.202101015 34057261

[108] F. Han, S. Luo, L. Xie, J. Zhu, W. Wei, X. Chen, F. Liu, W. Chen, J. Zhao, L. Dong, K. Yu, X. Zeng, F. Rao, L. Wang, Y. Huang, Boosting the yield of MXene 2D sheets via a facile Hydrothermal-Assisted intercalation. ACS Appl. Mater. Interfaces. **11**, 8443 (2019). 10.1021/acsami.8b22339 30697996 10.1021/acsami.8b22339

[109] M. Wu, Y. He, L. Wang, Q. Xia, A. Zhou, Synthesis and electrochemical properties of V2C MXene by etching in opened/closed environments. J. Adv. Ceram. **9**, 749 (2020). 10.1007/s40145-020-0411-8

[110] F. Bibi, A. Numan, Y.S. Tan, M. Khalid, Facile extraction of Mo2Ti2C3Tx MXene via hydrothermal synthesis for electrochemical energy storage. J. Energy Storage. **85**, 111154 (2024). 10.1016/j.est.2024.111154

[111] A. Numan, S. Rafique, M. Khalid, H.A. Zaharin, A. Radwan, N.A. Mokri, O.P. Ching, R. Walvekar, Microwave-assisted rapid MAX phase etching and delamination: A paradigm shift in MXene synthesis. Mater. Chem. Phys. **288**, 126429 (2022)

[112] F.N.M. Azlan, M.A.A.M. Abdah, Y.S. Tan, M.N. Mustafa, R. Walvekar, M. Khalid, Microwave-etched V2C MXene-activated carbon hybrid as a high-performance anode material for lithium-ion batteries. J. Energy Storage. **72**, 108620 (2023). 10.1016/j.est.2023.108620

[113] J. Zhu, J. Zhang, R. Lin, B. Fu, C. Song, W. Shang, P. Tao, T. Deng, Rapid one-step scalable microwave synthesis of Ti3C2Tx MXene. Chem. Commun. **57**, 12611 (2021). 10.1039/D1CC04989E 10.1039/d1cc04989e 34755720

[114] B. Fan, M.T. Ansar, Q. Chen, F. Wei, H. Du, B. Ouyang, E. Kan, Y. Chen, B. Zhao, R. Zhang, Microwave-assisted hydrothermal synthesis of 2D/2D MoS2/Ti3C2Tx heterostructure for enhanced microwave absorbing performance. J. Alloy Compd. **923**, 166253 (2022). 10.1016/j.jallcom.2022.166253

[115] D. Wang, C. Zhou, A.S. Filatov, W. Cho, F. Lagunas, M. Wang, S. Vaikuntanathan, C. Liu, R.F. Klie, D.V. Talapin, Direct synthesis and chemical vapor deposition of 2D carbide and nitride MXenes. Science. **379**, 1242 (2023). 10.1126/science.add9204. (acccessed 2025/06/05) 36952427 10.1126/science.add9204

[116] M. Öper, U. Yorulmaz, C. Sevik, F. Ay, N. Kosku, Perkgöz, Controlled CVD growth of ultrathin Mo2C (MXene) flakes. J. Appl. Phys. **131**, 025304 (2022). 10.1063/5.0067970

[117] J. Zhu, S. Zhu, Z. Cui, Z. Li, S. Wu, W. Xu, T. Ba, Y. Liang, H. Jiang, Solvent-free one-step green synthesis of MXenes by gas-phase selective etching. Energy Storage Mater. **70**, 103503 (2024)

[118] M. Lu, H. Li, W. Han, J. Chen, W. Shi, J. Wang, X.-M. Meng, J. Qi, H. Li, B. Zhang, W. Zhang, Zheng, 2D titanium carbide (MXene) electrodes with lower-F surface for high performance lithium-ion batteries. J. Energy Chem. **31**, 148 (2019). 10.1016/j.jechem.2018.05.017

[119] Y. Wang, C. Ma, W. Ma, W. Fan, Y. Sun, H. Yin, X. Shi, X. Liu, Y. Ding, Enhanced low-temperature Li-ion storage in MXene titanium carbide by surface oxygen termination. 2D Mater. **6**, 045025 (2019)

[120] J. Mei, G.A. Ayoko, C. Hu, Z. Sun, Thermal reduction of sulfur-containing MAX phase for MXene production. Chem. Eng. J. **395**, 125111 (2020). 10.1016/j.cej.2020.125111

[121] H. Li, A. Li, D. Zhang, Q. Wu, P. Mao, Y. Qiu, Z. Zhao, P. Yu, X. Su, M. Bai, First-principles study on the structural, electronic, and lithium storage properties of Ti3C2T2 (T = O, F, H, OH) MXene. ACS Omega. **7**, 40578 (2022) 36385825 10.1021/acsomega.2c05913 PMC9647848

[122] X. Pang, Z. Lv, S. Xu, J. Rong, M. Cai, C. Zhao, F. Huang, Ultra-conductive Se-terminated MXene Nb2CSe2 via one-step synthesis for flexible fast-charging batteries. Energy Storage Mater. **61**, 102860 (2023)

[123] B. Zhang, J. Zhu, P. Shi, W. Wu, F. Wang, Fluoride-free synthesis and microstructure evolution of novel two-dimensional Ti3C2(OH)2 nanoribbons as high-performance anode materials for lithium-ion batteries. Ceram. Int. **45**, 8395 (2019). 10.1016/j.ceramint.2019.01.148

[124] N. Xue, X. Li, M. Zhang, L. Han, Y. Liu, X. Tao, Chemical-combined ball-milling synthesis of fluorine-free porous MXene for high-performance lithium ion batteries. ACS Appl. Energy Mater. **3**, 10234 (2020)

[125] D. Gandla, Q. Li, Y. Zhou, Y. Yan, Z. Liu, J. Chen, D.Q. Tan, In-Plane mesoporous 3D Flower-Like Mo2Ti2C3Clx MXene anodes for Li-Ion batteries: from structure to performance. Small. **20**, 2404880 (2024). 10.1002/smll.202404880. (acccessed 2025/06/04) 10.1002/smll.202404880 39040006

[126] J. Gu, Q. Zhu, Y. Shi, H. Chen, D. Zhang, Z. Du, S. Yang, Single zinc atoms immobilized on MXene (Ti3C2Clx) layers toward Dendrite-Free Lithium metal anodes. ACS Nano. **14**, 891 (2020). 10.1021/acsnano.9b08141 31913604 10.1021/acsnano.9b08141

[127] L. Ma, Y.-K. Jiang, D.-R. Xu, Y.-Y. Fang, N. Li, D.-Y. Cao, L. Chen, Y. Lu, Q. Huang, Y.-F. Su, F. Wu, Enabling stable and Low-Strain Lithium plating/stripping with 2D layered transition metal carbides by forming Li-Zipped MXenes and a Li Halide-Rich solid electrolyte interphase. Angew Chem. Int. Edit. **63**, e202318721 (2024). 10.1002/anie.202318721. (acccessed 2025/01/20) 10.1002/anie.202318721 38294414

[128] Q. Jin, T. Zhang, Z. Dai, M. Zhao, L. Wu, L. Li, X. Zhang, X. Zhang, Tuning solvation behavior within electric double layer via halogenated MXene for reliable lithium metal batteries. Energy Storage Mater. **73**, 103837 (2024). 10.1016/j.ensm.2024.103837

[129] Z. Huang, T. Huang, X. Ye, X. Feng, X. Yang, J. Liang, S. Ye, Y. Li, X. Ren, W. Xiong, X. Ouyang, Q. Zhang, J. Liu, Constructing Methyl methacrylate/mxene artificial solid-electrolyte interphase layer for lithium metal batteries with high electrochemical performance. Appl. Surf. Sci. **605**, 154586 (2022). 10.1016/j.apsusc.2022.154586

[130] B. Zhang, W. Zou, Z. Ju, S. Qi, J. Luo, C.J. Zhang, X. Tao, L. Du, Separator engineering based on Cl-Terminated MXene ink: enhancing Li + Diffusion kinetics with a highly stable Double-Halide solid electrolyte interphase. ACS Nano. **17**, 22755 (2023). 10.1021/acsnano.3c07413 37931128 10.1021/acsnano.3c07413

[131] S. Moon, Y.H. Jung, W.K. Jung, D.S. Jung, J.W. Choi, D.K. Kim, Encapsulated monoclinic sulfur for stable cycling of Li–S rechargeable batteries. Adv. Mater. **25**, 6547 (2013). 10.1002/adma.201303166. (acccessed 2025/03/16) 24018843 10.1002/adma.201303166

[132] Y. Lin, J. Ticey, V. Oleshko, Y. Zhu, X. Zhao, C. Wang, J. Cumings, Y. Qi, Carbon-Nanotube-Encapsulated-Sulfur cathodes for Lithium–Sulfur batteries: integrated computational design and experimental validation. Nano Lett. **22**, 441 (2022). 10.1021/acs.nanolett.1c04247 34965149 10.1021/acs.nanolett.1c04247

[133] D. Zhang, S. Wang, R. Hu, J. Gu, Y. Cui, B. Li, W. Chen, C. Liu, J. Shang, S. Yang, Catalytic conversion of polysulfides on single atom zinc implanted MXene toward High-Rate Lithium–Sulfur batteries. Adv. Funct. Mater. **30**, 2002471 (2020). 10.1002/adfm.202002471. (acccessed 2025/01/21)

[134] L. Liang, L. Niu, T. Wu, D. Zhou, Z. Xiao, Fluorine-Free fabrication of MXene via Photo-Fenton approach for advanced Lithium–Sulfur batteries. ACS Nano. **16**, 7971 (2022). 10.1021/acsnano.2c00779 35466669 10.1021/acsnano.2c00779

[135] X. Zhang, Z. Ni, X. Bai, H. Shen, Z. Wang, C. Wei, K. Tian, B. Xi, S. Xiong, J. Feng, Hierarchical porous N-doped carbon encapsulated Fluorine-free MXene with tunable coordination chemistry by One-pot etching strategy for Lithium–Sulfur batteries. Adv. Energy Mater. **13**, 2301349 (2023). 10.1002/aenm.202301349. (acccessed 2025/02/03)

[136] Z. Wang, H. Jiang, C. Wei, K. Tian, Y. Li, X. Zhang, S. Xiong, C. Zhang, J. Feng, Ultrasmall CoFe bimetallic alloy anchored on Fluoride-Free MXene by One-Pot etching strategy for the Barrier-Adsorption-Catalyst functions of polysulfides in Lithium-Sulfur batteries. Adv. Funct. Mater. **34**, 2315178 (2024). 10.1002/adfm.202315178. (acccessed 2025/01/21)

[137] Y. Ren, L. Hu, S. Chang, Y. Ma, B. Wang, H. Wu, F. Li, Y. Yang, S. Tang, X. Meng, MXene-Bimetallic hybrids via mixed molten salts etching for Kinetics-Enhanced and Dendrite-Free Lithium–Sulfur batteries. Small. **20**, 2400068 (2024). 10.1002/smll.202400068. (acccessed 2025/01/21) 10.1002/smll.202400068 38593293

